# Partners in Recovery: an early phase evaluation of an Australian mental health initiative using program logic and thematic analysis

**DOI:** 10.1186/s12913-019-4360-2

**Published:** 2019-07-26

**Authors:** Steven A. Trankle, Jennifer Reath

**Affiliations:** School of Medicine, Department of General Practice, Western Sydney University, Campbelltown Campus, Building 30.3.18, Locked Bag 1797, Penrith, NSW 2751 Australia

**Keywords:** Partners in Recovery, Mental health, Program logic, Mixed methods, Australia

## Abstract

**Background:**

Mental illness is a leading cause of illness and disability and around 75% of people suffering mental illness do not have access to adequate care. In Australia, nearly half the population experiences mental illness at some point in their life. The Australian Government developed a National program called Partners in Recovery (PIR) to support those with severe and persistent mental illness. The program was implemented through 48 consortia across Australia. One of these was led by the Nepean Blue Mountains Medicare Local who adapted the program according to its specific local needs.

**Methods:**

We conducted an early evaluation of the PIR program in Nepean Blue Mountains (NBMPIR) using a program logic model (PLM) to frame the evaluation and complemented this with an additional thematic analysis. Participants (*n =* 73) included clients and carers, program management and staff of the Consortium and other partners and agencies, and clinical, allied health, and other service providers. Our PLM utilised multiple data sources that included document review, open and closed survey questions, and semi-structured interviews. Quantitative data received a descriptive analysis and qualitative data was analysed both in alignment with the PLM framework and inductively.

**Results:**

We aligned our results to PLM domains of inputs, activities, outputs, outcomes and impacts. The NBMPIR consortium implemented a recovery approach and provided greater access to services by enhancing healthcare provider partnerships. Our thematic analysis further described five key themes of collaboration; communication; functioning of PIR; structural/organisational challenges; and understanding of PIR approaches. Facilitators and barriers to the NBMPIR program centred on the alignment of vision and purpose; building an efficient system; getting the message out and sharing information; understanding roles and support and training of staff; building capacity and systems change; addressing service gaps; and engaging peers.

**Conclusions:**

Our study provided helpful insights into the coordinated management of complex mental illness. The NBMPIR’s focus on partnerships and governance, service coordination, and systems change has relevance for others engaged in this work. This PLM effectively mapped the program, including its processes and resources, and is a useful framework providing a baseline for future evaluations.

Full report available at https://researchdirect.westernsydney.edu.au/islandora/object/uws:33977/

**Electronic supplementary material:**

The online version of this article (10.1186/s12913-019-4360-2) contains supplementary material, which is available to authorized users.

## Background

The World Health Organisation states that mental illness is the leading cause of ill health and disability worldwide [1]. Moreover, health systems around the world have not responded adequately leaving 75% of those suffering mental illness without treatment [1, 2]. Almost half the Australian population (45.5%) experiences a mental health disorder at some point in their lifetime and 20% of the Australian population aged 16–85 years have experienced a mental disorder in the previous 12 months [3]. There can be far-reaching impacts on those affected, their families and carers, and for the Australian community [4].

Mental illness is complex with many factors influencing its expression and impacting on management [5–7]. Successful management requires individual and systematic approaches that incorporate biological, social and psychological perspectives [8, 9]. These approaches are recommended to include recovery-based, consumer-driven, locally specific approaches focussed on empowerment [10–13], and coordination and integration of the multiple services required to meet consumer needs [5, 14]. However, barriers to recovery based care models have been identified including management conflicts [15], difficulties accurately measuring consumer and system outcomes [5, 10, 12, 14] and need for a commonly understood conceptual framework to guide practice [10, 12, 16], challenging the expertise of service providers [12, 17], and failure to value the experiences of street level workers [15, 18] and consumers [16, 19, 20].

Australia has a fragmented health system that is funded by federal, state and local governments [21, 22]. Medicare is Australia’s publically funded universal health care system and provides access to some health services at low or no cost. While public hospitals are managed by the state, most out of hospital and primary and allied health care services are delivered by private providers [23]. Prior to the implementation of a nationally focussed approach, mental health services often failed to adequately address the needs of those with more complex and chronic problems and were not enabled to address social service needs or to “join up” the care across jurisdictions [24].

Following the launch of a national mental health policy [4], the Australian Government developed a national framework for recovery-oriented mental health services [25]. As part of this framework, the Partners in Recovery (PIR) program was implemented nationally in late 2012 and aimed to provide better support to people with severe and persistent mental illness, including for their carers and families, using a collaborative, coordinated, and integrated approach [26].

Forty-eight regional PIR consortia were funded nationally. Each drew on the strengths and resources of the local consortium partners to provide services meeting the needs of the region [24, 27]. Some consortia recruited clients from hospitals and other mental health services while others recruited more from the community including from Aboriginal health services [28–30]. Partners in Recovery was defined by a facilitation approach intended to empower clients rather than a case management approach [31]. Support Facilitators were engaged to provide client liaison and education and to coordinate client services [22].

Evaluations of other PIR programs have noted the central role of the support facilitator [22, 27] and how the role is adapted in different contexts. An effective organisational hierarchy was also considered important to allow the support facilitator role to develop within the program [22]. Although PIR programs have reduced unmet needs and enhanced mental health recovery [29, 32], some have highlighted challenges in reaching certain populations such as Aboriginal people [28]. Some needs such as daytime activities and accommodation were also not always met [28]. Evaluation approaches were found to be inconsistent in some programs limiting the evidence base for these programs [28].

The Nepean Blue Mountains Medicare Local (NBMML), currently known as the Nepean Blue Mountains Primary Health Network, was the Lead Organisation responsible for implementing PIR in the Nepean Blue Mountains area, west of Sydney. The NBMML was a not for profit primary health care organisation funded by the Australian Government seeking to improve primary health care in its local region. The Nepean Blue Mountains region has a diverse population with wide ranging health needs [33, 34] including 3.7% who identify as Aboriginal and Torres Strait Islander and 22% from culturally and linguistically diverse (CALD) backgrounds, most of whom were born overseas and speak English as a second language [35]. In establishing Nepean Blue Mountains Partners in Recovery (NBMPIR), the NBMML joined with partner organisations including the Nepean Blue Mountains Local Health District, Family and Community Services, RichmondPRA, Uniting Care Mental Health, and Aftercare, as well as consumer representatives to form a consortium. Most consortium organisations were already involved in mental health in the region. This consortium also collaborated with numerous government and non-government (NGO) non-consortium partners including community organisations and other service providers to refer clients to the program and draw on their services. These included Personal Helper and Mentor Support (PHaMS), Housing and Accommodation Support Initiative (HASI) and Centrelink (Australian welfare and employment agency). The key objectives of NBMPIR were focused on:

- Partnerships and governance;
- Service coordination; and
- System change

Our research examines achievement of these key objectives in a region with diverse population demographics. Unique to our research is use of a comprehensive evaluation approach including document review, survey and interviews, framed by a program logic model, and supplemented by an inductive thematic evaluation.

### Research aims and objectives

The aim of our research was to conduct an early evaluation of the NBMPIR program, 2 years from commencement of funding.

Our local evaluation of NBMPIR complemented a national evaluation of Partners in Recovery [36] and reflected on the effectiveness of the locally implemented program at an early stage according to its key objectives, including its facilitators and barriers, in order to provide learning for future operations. In this paper we report the findings of our evaluation and reflect on the value of a program logic model to frame our evaluation. This research is presented in greater detail in the full report [37].

## Methods

### The researchers

Dr. Steven Trankle (PhD) has a background in psychology and Professor Jenny Reath has clinical expertise in General Practice medicine. Both researchers have experience in health services research, and with multiple methods, and are engaged as researchers and educators.

### A program logic model framework

**Fig. 1:**
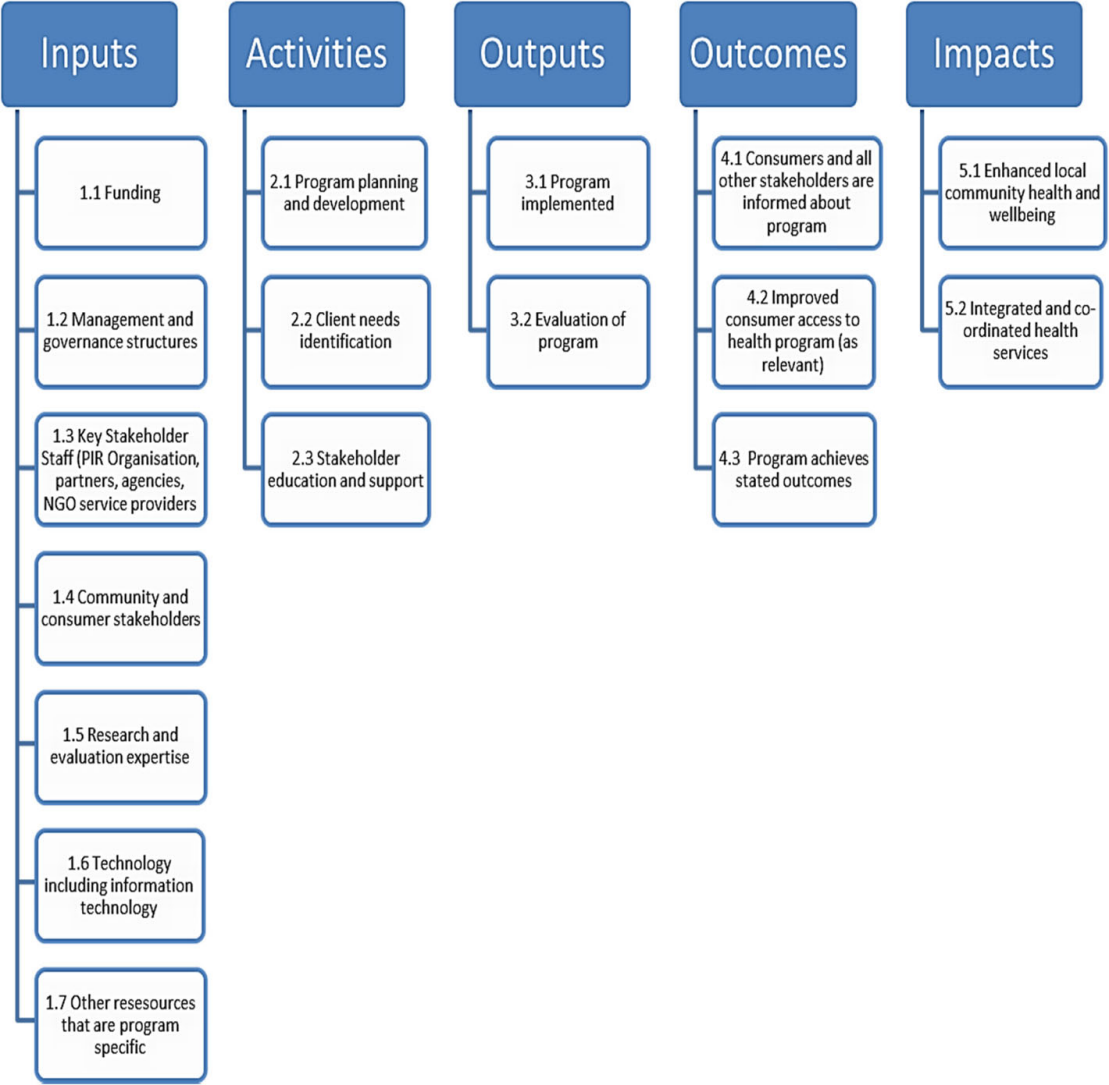
Program Logic Model of Nepean Blue Mountains Partners in Recovery

We engaged a Reference Group of key stakeholder representatives from the NBMML to advise on the planning and implementation of the evaluation. After reviewing the literature, we consulted with the Reference Group and other key stakeholders to develop a program logic model (PLM) of the NBMPIR and assigned indicators for the program’s inputs, activities, outputs, outcomes and impacts (Fig. 1). Our evaluation aimed to measure achievement against each of the assigned indicators (Additional file 1).

Use of PLMs in evaluation is said to “provide learning opportunities, better documentation of outputs and outcomes, and shared knowledge about *what works* and *why*” [38]. Program logic approaches to evaluation have been described as helpful in understanding and evaluating complicated and dynamic systems [39, 40] including in mental health domains [41, 42] and also in primary health systems [43–45]. A PLM approach was particularly suited to evaluating the NBMPIR in a demographically diverse local community with complex needs.

### Ethics approval

We received ethics approval from the following Human Research Ethics Committees:

- Western Sydney University Human Research Ethics Committee (H10697); and
- Nepean Blue Mountains Local Health District Human Research Ethics Committee (14/49).

This research also received approval from the Nepean Blue Mountains Local Health District Scientific Advisory Committee.

## Data collection and analysis

We used a mixed methods approach [46, 47] collecting and synthesising data from the following sources:

- Review of policy, operational and reporting documents of the NBMPIR;
- Quantitative and qualitative survey data; and
- Qualitative interview data.

Our reporting of qualitative data aligns with the COREQ guide [48].

### Document identification and analysis

Information sources were identified in consultation with the Reference Group to measure indicators related to the NBMPIR and included a range of governance and organisational documents such as Memoranda of Understanding, Service Level Agreements, terms of reference, meeting minutes, and relevant mandated reporting documents to Department of Health such as quarterly performance reports, as well as other reports of activities and plans (Additional file 2).

### Survey

The survey included both Likert-based questions and open-ended questions aligned to the PLM indicators (Additional file 3). Questions centred on participant roles and their experiences and perceptions of implementing the NBMPIR program. It was circulated in on-line and paper-based formats.

### Interviews

A semi-structured interview guide was developed to further assess the PLM indicators (Additional file 4). This provided in-depth responses to those issues canvassed in the survey, and an opportunity to explore perceptions particular to the individual [47]. The guide was piloted in the first 5 interviews to ensure it adequately covered each stakeholder group. Interviews were between 30 and 60 min in duration and conducted one-on-one and face-to-face by ST at NBMML or health care provider offices. All interviews were audio-taped and transcribed verbatim by an independent transcription service. Transcripts were then checked by ST for accuracy. Participants were also offered their transcripts to check accuracy.

### Data Analysis

We used two approaches to analysis of the data, an initial deductive approach using the PLM as a theoretical framework for data analysis and a second thematic analysis of qualitative survey responses and interview data.

In the framework analysis we mapped our data sources against each element of the PLM [38, 49]. In analysing the data collected we used a descriptive approach to report responses to closed survey questions and integrated qualitative information from free text responses and interviews as well as data from our document review.

The second inductive approach, utilised a separate thematic analysis of qualitative responses according to established protocols [50–52] using N-Vivo 10**®**. We explored emergent themes across all survey and interview data and noted discrepant responses. Any unrepresentative quotes were identified as such. The initial five interview transcripts were coded independently by ST and JR and a third researcher (MK) and we then also cross-coded each other’s. We discussed our analysis and agreed on potential themes which provided a framework for further interview analysis. After interviewing concluded, we iteratively read and re-read each transcript and coded until we achieved saturation of the main themes. We then reviewed the coding frame again in order to confirm coding and the positioning of subthemes within main themes (Additional file 5). Lastly, we provided a selection of powerful and compelling quotes from all participants to represent the themes.

### Participants

Participants were purposively selected across a variety of roles within PIR rather than on the basis of demographic characteristics. This ensured we adequately sampled from the available stakeholders with experience of the program. They included clients and carers, management and staff of the NBMML, Consortium and other partners and agencies, and a range of clinical and allied health, and other service providers. NBMPIR recruited participants and provided them with an ethics approved letter of invitation, information and consent sheet, and an online survey link or a mailed paper copy as preferred. To protect confidentiality NBMPIR did not know who completed the survey as this was administered by the researchers. Participants were recruited for interviews through an invitation in the survey requesting their contact information. Interview participants were then contacted directly by ST to schedule their interview. Signed consent was obtained for interviews and hard copy surveys. The information sheet provided by NBMPIR also included information about the researchers. Participants did not know the researchers prior to the study. No participants withdrew from the study.

## Results

### Participants

A total of 73 participants completed the survey and 17 of those participated in an interview (Table 1). Community representatives worked in consumer advocacy and support roles, with some being employed by other mental health services, and included disability advocates, consumer representatives, and local government community development officers. Consortium partners and staff were involved in an administrative capacity and not as health care providers. The Support Facilitator role provided liaison between clients and services.

**Table 1:** Survey and interview participation

Participant	Role	Survey	Interview
Board/Management/Staff PIR and consortium/other non-consortium partners	Managers – NBMML (Lead Org), Consortium & Partner Agency	7	5
	Staff - NBMML	7	1
	Staff - Consortium Partner	3	1
	Staff – HASI^a^	1	
	Staff – PhaMS^b^	2	
	Community Residential Rehab Program - Staff	1	
	Support Facilitator	12	2
Health Care Providers	Psychiatrist	1	1
	Allied health	7	
	Case Manager	1	
	Community Service Worker	2	1
	Mental Health Nurse	1	1
	Counsellor	1	
	Support/Social Worker	3	1
	GP	1	
	Other	1	
Clients		11	1
Carers		4	1
Community Representatives		7	2
Totals		73	17

^a^Housing and Accommodation Support Initiative

^b^Personal Helper and Mentor Support

### Data analysis

Our results are presented in two sections reflecting our use of two approaches to analysing the data we collected. We provide a PLM framework analysis first and then a separate thematic analysis.

### Program logic model - framework analysis

In this analysis we describe how the documents, survey and interview responses address each of the PLM indicators in terms of program Inputs, Activities, and Outputs and, in a more limited way, Outcomes and Impacts (Additional file 1). Findings related to these longer term program effects will require later evaluation.

### Inputs

Program logic model inputs are the “human, financial, organizational, and community resources a program has available to direct toward doing the work” [38]. Key inputs identified in our NBMPIR PLM were funding, management and governance structures, staffing, community and consumer stakeholders, and information technology.

The documentation we reviewed identified key areas of expenditure and amounts disbursed and all respondents agreed funding was adequate. At this early stage of implementation, difficulties were identified with providing partner organisations with access to this funding.

Clinical and corporate governance protocols had been established and were well documented, with most survey respondents agreeing that organisation and management of PIR was open and transparent. A client management information system (CMIS) called PENELOPE recorded client information and progress through the program.

Interviewees described a collaborative respectful approach with one interviewee commenting: *“there is a strong and robust relationship within the Consortium working towards common goals for the people that we work with” (NBMML staff).* However, one Support Facilitator noted some instability, commenting: *“there’s been lots of chops and changes in the [Lead Organisation] management”.*

Roles and responsibilities of key service personnel were identified in reviewed documents and these informed work practices Surveyed NBMML PIR staff and management agreed or strongly agreed that they had clear job descriptions (9/10) and that their practice matched their defined roles (8/10) and consortium staff similarly agreed their PIR roles were clear (6/8).

In terms of community and consumer engagement, community-based NGOs were engaged in the Consortium, and client and carer representative positions were filled. Community promotion of PIR was well attended through press releases, newsletters and community fora across all local government areas**.**

All clients (11/11), and most carer (3/4) and community representative (5/6) survey respondents agreed or strongly agreed that PIR sought consumers’ views and most agreed or strongly agreed that they had sufficient opportunity to provide feedback aimed at improving NBMPIR.

Information technology was a key input and training was provided for staff particularly in use of the CMIS - PENELOPE. Half (5/10) of the NBMML management and staff survey respondents agreed that IT and training met their requirements and most agreed that IT assisted communication, and was used efficiently. Interview data supported these findings with staff and relevant stakeholders reporting satisfaction with IT and the training provided: *“I’m finding PENELOPE really helpful and I think it’s great. I think it’s really, really good and the [IT] support we’re getting now is really great” (Support Facilitator).*

### Activities

Program logic model activities are the “processes, tools, events, technology, and actions that are used to bring about the intended program changes or results” [38]. Performance indicators in our PLM were related to program planning and development, client needs identification, and stakeholder education and support.

Program planning and development was attended through establishment of a commonly understood framework of language; consultation with service providers, community and researchers to inform ongoing program implementation; development of subprograms; and implementation of strategies to inform stakeholders of the program.

A clearly operationalised recovery focus informed key documents such as the PIR service manual and aligned strongly to national PIR policy documents and the recovery language guide from the Mental Health Coordinating Council. Half of the health care providers (8/16), most staff and management from the NBMML (7/10) and consortium partners (13/16), and all community representatives and other non-consortium partner staff agreed the framework of language assisted in building an understanding of PIR. A surveyed consortium staff member said: *“Language is consistent and clear. Definitions and meanings are clear”* while, in contrast, a NBMML manager noted: *“a shared use of language has been established and is important but it isn’t necessarily a shared understanding”.* Concepts of recovery appeared to be understood by most with descriptions provided by interviewees such as: *“empowerment – having clients say what their definition of recovery is” (Community worker).* However, implementing recovery based approaches was sometimes challenging: “*it’s something that we’re really struggling to build in with that kind of overarching medical model” (NBMML staff).*

Monthly Consortium and Support Facilitator Working Group meetings, which included consumer and carer representatives, fostered collaboration and facilitated consultation with key stakeholders. Most clients (3/5), carers (2/2) and community representatives (3/5) surveyed agreed PIR engaged them in planning programs. This contrasted with health care providers, most of whom disagreed with this statement (8/15). Though a communication strategy had been developed by NBMPIR, at times external stakeholders found the communications inadequate: *“they first introduced themselves last year, maybe mid last year, but then we didn’t hear anything further from them” (Consortium staff).* Most respondents noted strong common interests and goals among Consortium members through comments such as: *“When we’re raising things at the Consortium, nearly everyone’s saying yes, that’s an initiative we have to fulfil, that’s a KPI for us - seems to be synergy there” (Consortium manager).*

Subprograms focused on systems change were being developed such as Capacity Building, enhanced Consumer/Carer engagement, Smoking Cessation and Physical Fitness and Co-location of NBMML staff with Consortium and other provider organisations [37]. Discussion about systems change occurred with senior management of all stakeholder organisations. Interviewees spoke about their understanding of systems change with collaboration a common theme: *“Just getting people to work together. No longer that silo effect” (Support Facilitator).*

Promotional strategies were tailored to suit different referrers and a website and quarterly newsletters provided information. Within 2 months a rapidly growing client waiting list required temporary suspension of promotion.

Client needs and eligibility were identified at intake through the Camberwell Assessment of Need Short Appraisal Schedule (CANSAS). Interviewees noted that CANSAS did not capture enough information and also described contractual constraints:

*We didn’t have as much freedom as we might have had in relation to that because of the requirement to use CANSAS …it’s not really a tool with the consumer in mind. There’s nothing that says this is how people’s lives have changed (NBMML manager).*

An individualised recovery plan was developed for coordinated support, and periodic assessments undertaken to provide information on client progress. Interviewees highlighted the importance of considering context: *“I think that people are still finding out what the local needs and conditions are. I think we’re trying to be fluid enough to keep up” (NBMML staff).*

Tailored stakeholder education and support was provided and a comprehensive Education Plan had been finalised at the time of our evaluation. Most of those surveyed agreed that support and education were satisfactory, although some health care providers disagreed (6/16) or responded neutrally (7/16) to this question. Survey respondents across stakeholder groups contrasted with interviewees who described the support and education provided to staff and other stakeholders as inadequate. Comments included: “*I was thrown in at the deep end” (NBMML staff),* and *“I have received nil education and support from PIR workers” (Health care provider).* Health care providers were particularly varied in their views about education and support with one reporting*: “We also had quite a lot of written material around what Partners in Recovery does”* whilst another suggested*: “a flyer and referral information would probably be quite helpful”.*

### Outputs

Program logic model outputs are the “direct products of activities including new resources, services and programs delivered by the NBMML” [38]. Performance indicators in our PLM related to how the program was implemented and operating, including staff orientation and support, client intake, referral pathways, stakeholder engagement and satisfaction, and perceived efficiency of PIR operations and to evaluation of the program.

Reviewed documents revealed that most staff were appointed as planned and all relevant staff including Support Facilitators were trained in CMIS and the PIR Mental Health Care Coordination strategy. Some reported having existing skills: *“I’ve been involved in homelessness provision for a long time. So when PIR came along, it felt natural with my own personal professional journey” (Consortium staff).*

The majority of clients were referred from community based mental health services and other community services. Client intake recorded in the CMIS indicated that demand was higher than expected in all but one more geographically isolated area of NBMML. This illustrated that intake pathways were working. However, it was also noted that people who were homeless or destitute may not be accessing the program (NBMPIR Annual Activity Work Plan 2014–2015). By the end of 2015 all planned Support Facilitators had been engaged and the waiting list was reduced.

Interviewees commented on the effectiveness of referral pathways*: “It’s quite easy. The referral pathways are something that, I think, the original information explains very well (Consortium manager)”.* Support Facilitators considered these pathways a result of their work *“the more Support Facilitators there’s been the more referral pathways that are being created”,* although some health care providers were unsure of referral pathways: *“I really don’t know, to be honest”*.

Although interviewees reported limited stakeholder engagement in the program at this early stage, their experiences where this occurred were generally positive. One interviewee commenting on PIR staff noted: *“people were really motivated and enthusiastic about getting everyone together. I think that was really good” (Carer).* A health care provider with a client who was very reluctant to engage praised the Support Facilitator saying: *“he’s great, really great. My client has a really good rapport with him and he’s quite aware of her needs, her limitations and things like that which has been really helpful”*. One client highlighted the importance of engagement: *“I think it depends on the amount of input I put in as well. So yeah, I’m satisfied with my engagement so far”.*

Although most PIR staff and management in non-consortium partners agreed that implementation of PIR in the early stages was effective (4/5), responses from those more involved in the daily operations were less positive. At interview a consortium manager involved from the beginning commented: *“It was effective in bringing the partners together in the one place, bringing senior figures together and engaging in each other’s strategies, having that sense of being in it together”.* Whilst the partnerships were attended, provision of services appeared to take longer with another consortium manager commenting: *“The services side probably took a bit longer to set up than expected”.*

A key PLM indicator related to efficiency and cost effectiveness and reports indicated that the budget was not fully expended at the time of our evaluation. Some interviewees identified difficulties accessing funds from NBMML: *“for the first eight or nine months we had no access to funding so it was really difficult” (Consortium manager).* These early challenges with disbursement of funds may have impacted on survey responses indicating that only 50% of NBMML respondents believed PIR was efficient and cost effective.

Evaluation of the program was another key PLM indicator and included active monitoring of the program and use of the data to inform further development.

CMIS data provided detailed information about consumer recovery pathways and indication of where gaps were occurring such as for homeless people. However, there was little information about Aboriginal and Torres Strait Islander and CALD consumers use of the program (NBMPIR 6 Monthly Performance Report - Qualitative). Interviewees reported that evaluation activities were common within PIR and were often focused on specific stakeholder groups: *“just about to implement a three-month consumer feedback form – to be given to all consumers” (NBMML staff).*

Evaluation informed future program development with documents describing ongoing review of operations, including through use of CMIS data (NBMPIR 6 Monthly Performance Report - Qualitative). These reviews resulted in improvements such as revised referral forms and development of a waitlist policy. Interviewees described this program development: *“With PENELOPE data as well, that would assist us in moving forward to tweak and - or change what we’re doing” (NBMML manager).*

This evaluation focus was noted by survey respondents apart from health care providers and interview data reflected these views. One consortium manager stated: *“All quality improvement initiatives are evaluated and measured”* with a health care provider reporting: *“I have not been asked for feedback by PIR workers or management before completing this survey”.*

Most stakeholders were satisfied with their experience of evaluation. Commenting on the current Western Sydney University evaluation, one health care provider said: *“like this evaluation process? Which is good, excellent, I think that’s great, I haven’t seen that happen before with any other service. That impresses me”.* Similarly, a Support Facilitator commented: *“I quite like the extensiveness of who is giving feedback and how the program is being evaluated”.*

### Outcomes

Program logic model outcomes are the “specific changes in program participants’ behaviour, knowledge, skills, status and level of functioning” [38]. Although this was an early evaluation of PIR, some outcomes were described in accordance with PLM indicators including stakeholder knowledge of the program, consumer access to PIR services, program achieving its stated aims in terms of coordinated care, health status and functioning of consumers, and improvements in service provider knowledge, skills, functioning and collaboration.

Ten percent of referrals to NBMPIR were from clients and carers suggesting some level of awareness about the program amongst these stakeholder groups (NBMPIR 6 Monthly Performance Report - Qualitative) which was also supported by survey responses and in interviews. One carer said: *“I think it’s good. I never knew anything about it. The mental health team rang me and asked me if I would go in it and I did. The information is good”,* while a community worker similarly noted: *“My knowledge of the program has changed from being kind of hypothetical to being practical - satisfied for sure”.* Health care providers also indicated their increased awareness: “*I’ve actively made referrals to PIR to try and address the identified need for the client. So that’s changed”* although one health care provider commented: *“I’ve been trying to find out more but the more I find out, the more confused I am. So it hasn’t changed, I guess”.*

Consumers could better access PIR services according to all staff and management of stakeholder partners, and most health care providers (8/15). Clients (7/8) and carers (2/4) also agreed that PIR had assisted them in getting the right services although survey respondents described a need for widespread promotion of NBMPIR to increase consumer access to PIR services: *“from what I have seen so far it’s about increasing the public awareness of PIR; other service providers and LHD’s [Local Health Districts] need to be advised on what PIR is about” (NBMML staff).*

Interviews with consumers and carers revealed variation in access to PIR services. One client reported: *“Directly through my case manager. And that’s pretty much through a phone call,* whilst a carer had a contrasting experience: *"I just found that Nepean [PIR] took a long time to get back to me”.*

The program was widely observed to have achieved its stated aims with most respondents considering PIR effective in coordinating support for people with severe and persistent mental illness. Organisational staff commented *“It’s certainly effective from the point of view of putting the person in the driving seat of their recovery” (Consortium manager)*, and *“It is effective and that’s not just evidenced with how well our consumers are doing, it’s also evidenced within the relationship of the consortium and other NGOs - we’re able to build synergies with other organisations, it’s brilliant actually” (NBMML staff).* A client also reported how PIR had helped him*:*

*PIR funded additional sessions with my psychologist when I had a break down. Having that support kept me from self-harm or other destructive behaviours that have been a coping mechanism for me in the past. They also helped me find a new house, which is huge. My worker did all the stressful things which let me focus on the day to day. I am grateful.*

Others however described reservations: *“Inconsistent. I’ve had some fabulous experiences with Partners in Recovery but there’s been some not so good ones” (Health care provider).*

Consumer health status and functioning had improved through engaging with PIR according to most surveyed clients (5/8) and carers (2/3). Interviewees generally commented positively on their interaction with PIR: *“They have helped me significantly in many ways. I have felt supported and safe with my PIR worker” (Client).* “*My brother is already showing signs that Partners in Recovery have helped him greatly” (Carer).* Carers also noted reduced burden: *“It gives me a break from being a carer” (Carer).*

Most clients (5/8) and carers (2/3) agreed they had more hope for their future health and functioning as a result of PIR. One client said: *“PIR helped me gain independence by living alone. I now feel more confident about my abilities to live normally without support”,* while another offered to engage in peer work by helping others: *“…I’ve actually spoken with my case manager about trying to do some volunteer work myself”.*

Provider knowledge, skills and level of functioning had improved for most surveyed consortium (8/13) and non-consortium partner staff (3/4). However, this was different for health care providers who equally disagreed (7/14) or were neutral (7/14) with this survey statement. Interviews contrasted with these responses with one health care provider describing self-improvement: *“Modelling of good care coordination has been something that I’ve picked up personally; and every skill of mine - that’s improved through my contact with Partners in Recovery”.*

New and more effective partnerships helped staff to meet the needs of consumers. Most surveyed consortium (10/13) and non-consortium partner (3/4) staff and management agreed that PIR had assisted them with partnership engagement. Although health care providers mostly disagreed (8/15) that PIR had assisted them in this way, qualitative survey feedback was sometimes positive:

*It certainly extends your knowledge of all of the different services available - aged care providers, and disability services, and all kinds of other areas that we don’t get a lot of training and don’t necessarily have a lot of contact with (Health care provider).*

### Impacts

Program logic model impacts refer to the “fundamental changes occurring in organizations, communities or systems as a result of program activities” [38]. The indicators described for this domain related to improved community health and well-being, and better integration and coordination of health services. At the time of our evaluation, we did not expect to find evidence of impacts. However, survey and interview data suggested some early and potential program impacts.

To understand enhanced local community health and well-being, we asked about improvement in access to required services and supports. Most surveyed community representatives (3/5), clients (6/8) and all carers agreed that PIR has resulted in sustained improvement in access and non-consortium partner staff and management also agreed with this statement (3/4). Interviewees provided examples: *“Being put in touch with the organisation has provided me with accommodation. They provided a few other services as well which I have occasionally accessed” (Client),* and from a health care provider: *“I can only speak about this one case. The client is now able to advocate for themselves, the client now has more agency and understands how to work with the services. Generally speaking, I think it’s working really well”.*

Our survey also explored referral pathways for consumers of CALD and Indigenous backgrounds, with 50–60% participants across all stakeholder groups responding neutrally to this question. Qualitative feedback suggested this would be a later focus of the program: “*CALD communities have not been a major focus as yet of NBMPIR probably due to the relatively small percentage of this group in regards to the rest of Sydney” (NBMML manager),* and would require employment of CALD and Aboriginal staff: “*We have NO CALD staff and NO Indigenous identified staff, how are we meant to engage with these communities without this” (NBMML staff)?*

We looked for evidence of integrated and coordinated health services through improved team work and better care coordination, as well as improved client access to housing, employment, education and social activities, and evidence of clinical and community support services operating according to a community-based recovery model.

Improved team work was seen with most surveyed NBMML (8/10), consortium (7/13) and other non-consortium partner staff and management (3/4) agreeing that PIR had assisted them in networking with other stakeholders in order to respond to consumer needs. Health care providers also described improved team work in their interviews: *“In the past it’s been hard to get everyone working together, it appears to be a huge improvement”.*

However, at interview perceptions varied and some were critical about this aspect of the program: *“We’re getting quite a lot of resistance from people saying, you know, it’s not your role, it’s not my role or you shouldn’t be doing that or in some cases they’ve said we’re not going to refer to you” (Support Facilitator)*. Interviewees also described how siloed and entrenched practices were difficult to change: *“Changing the attitudes of people who have been in the industry for 20 or 30 years and have been trained and practiced with that medical model for so long, it’s very difficult to shift” (Community health worker).*

Partners in recovery had improved consumer access to integrated services that addressed multiple needs according to most surveyed staff and management of the Lead Organisation (8/10), consortium (7/13) and other non-consortium partners (3/4), as well as clients (7/8) and carers (3/3). Community representatives provided a neutral response neither agreeing nor disagreeing as to any improvement in this area (5/5). Interviews revealed difficulties as described by one community worker: *“We get a lot of homeless people come in here in crisis, so we’re dealing with crisis situations,* and a carer said: *"It’s social I need because he can’t work and there’s no way he can live by himself. They’ve got to have social interaction”.*

The recovery model of care was understood well by most surveyed staff and management of the Consortium (12/13) and other non-consortium partners (4/4), and health care providers (11/14). However, interviews suggested that recovery based services were not universally accepted: “*It’s mixed- there’ll be some pockets where the focus is more clinical than recovery focused” (NBMML Manager).* One respondent described a pro-active response to this issue: *“Part of our systems change is addressing that and at least providing modelling for what recovery looks like” (NBMML staff),* and early signs of change were noted by others:

*Health in the last couple of years has made a really big effort to kind of change the culture of the services that we provide, and really kind of try to build in that recovery-oriented focus. But at the same time health is also a beast that moves very, very slowly. And it is very difficult to change that culture (Health care provider).*

### Thematic analysis

To complement the PLM analysis, we conducted a separate thematic analysis on the 17 transcribed interviews and on the open ended survey responses provided [52]. This enabled us to deepen our understanding of the NBMPIR program. Five key themes were identified, specifically: collaboration; communication; functioning of PIR; structural/organisational challenges; and understandings of PIR approaches. Each of the key themes was further elaborated by a range of subthemes which are described below.

#### Collaboration

We found that collaboration improved among different providers as the program became more established, although not all interviewees agreed this was the case. The NBMML was, however, considered instrumental in reducing siloing (Table 2).

**Table 2:** Collaboration

Subtheme	Representative Quotes
Working together and reconciling differences	• Once they [NBMML] got up and running in those roles, we then had a lot more to do with each other in terms of engaging all the stakeholders with a range of different things (Consortium Partner). • We all had dispute resolution issues, where management have come in and we had clients and team leaders and clinicians in kind of big round table meetings, talking about who is doing what (Consortium Partner).
Non-collaboration	• It’s been pretty directive [from NBMML] – it’s not been collaborative really (Service Provider).
Working against siloed provision of care	• They [NBMML] are attempting to get all the people that are not communicating effectively and off in their own little silos doing their own programs, to work together and be more collaborative (Health care provider).

#### Communication

Communication was identified as key to effective operation of PIR. Communication was improving across provider networks and with consumers who valued NBMPIR. Program staff were providing feedback to enhance the program and the consumer voice was regarded as crucial including in planning stages, however, promotion of NBMPIR did not reach all stakeholders initially (Table 3).

**Table 3:** Communication

Subtheme	Representative Quotes
Organisational communication	• I’ve seen a lot of improved communications and it’s easy to call Partners in Recovery and ask questions (Consortium Staff).
Consumer contact	• Yeah, just out of the blue, phone calls just to see how things are and following up basically (Client).
Feedback and consultation informing PIR	• So I’d offer feedback to them just if there were little issues regarding what was available or gaps in what a service can provide and stuff like that (Consortium Staff). • Please involve consumers and their representatives earlier in the bureaucratic process of PIR planning, operations and delivery and please remember in policy, operations and action that consumer needs are the goal over bureaucratic needs (Consumer Rep).
Promotion of programs	• They give me flyers and I certainly promote their service at any community event that I have (Community Service Worker) • One thing that springs to mind is we did not have a launch here (Support Facilitator).

#### Functioning of PIR

Use of recovery language and practice using a recovery framework were becoming more common. Consumers were increasingly involved in the program’s implementation and considerable improvements were noted in their mental health status. Referrals into the program increased rapidly from a wide range of sources especially through Support Facilitators who were developing new service partnerships, however a range of service gaps were identified (Table 4).

**Table 4:** Functioning of PIR

Subtheme	Representative Quotes
PIR working well	• I got the first sense of the partnership genuinely working pretty early on in my involvement when everybody around the table was using recovery oriented language (NBMML Manager) • We have a consumer worker who once was a heroin user and has a diagnosis of schizophrenia. She’s in regular employment and has been well for some period of time (NBMML Manager) • More person-focused and consumers are encouraged to have a voice (Consortium Manager) • Keep helping because… a lot of people need this service. You are a God send to us (Carer)
Work in progress	• It takes a long time to change how people approach mental health. Recovery oriented practice is starting to happen. New grads are coming through, and starting their careers with that mindset, that’s exciting (Community Support Worker).
Access to the program	• It varies enormously from place to place. One area mental health team is really enthusiastic and referrals are pouring out of them…another area mental health team gives us next to no referral (NBMML Manager). • Clients who were referred to other agencies like the housing programs in the area, when their wait lists are too full, are being referred on to PIR (Community Service Worker). • I think that the more Support Facilitators there’s been, the more referral pathways that are being created (NBMML Staff).
Challenges of service gaps	• Housing, social needs. Those are the two highest and, I’d say, third or fourth were getting a job (NBMML Manager). • We’d really like to be offering more clinical groups, offering more acceptance and commitment therapy groups, DBT^a^ groups, working closely with drug and alcohol service to really, make an impact on substance use presentation (Community Service Worker).

^a^Dialectical Behaviour Therapy

#### Structural/organisational challenges

Structure and organisational challenges were frequently mentioned by respondents. In this early phase, bureaucratic processes and lack of clear guidelines made accessing resources difficult while IT and communication systems were considered inefficient. To counter a perceived lack of provider education, NBMPIR co-located Support Facilitators across the provider network. The need for long term approaches was identified to achieve sustained improvements in consumer outcomes and to change entrenched attitudes averse to systems changes and recovery approaches (Table 5).

**Table 5:** Structural and organisational challenges

Subtheme	Representative Quotes
Difficulties negotiating decision making processes	• I’m not totally across all the bureaucracy yet and the unwieldy bureaucracy is one of the difficulties (Consumer rep)
Difficulty accessing and using resources efficiently	• I think the use of information technology and the form of databases is as much an obstacle as an asset…nothing is purpose built (NBMML Staff). • We need a direct line to Housing, we need a direct line to Centrelink – we’re sitting for 45 min on the phone (NBMML Manager).
No overriding direction	• It’s been really difficult because you don’t have any guidelines. We don’t have any – this is what we’re doing and this is the way it should be (NBMML Staff).
A need for education, training and support	• When we were having problems with our local mental health service, it was evident that education hadn’t filtered down to the ground level, and that’s why we’re getting so much resistance (Support Facilitator). • …within the co-location program. We’re training Centrelink staff in first-aid and how to work with people with a mental illness (NBMML Staff).
Revert to default positions	• I think they [Clinical Services] see us as possibly a threat in some way, or it’s harder for them to change their mindset, being so clinically focused (Support Facilitator).
Risk Aversion	• It’s [PIR] seen as something really alien and different and Medicare Locals in health related coordination are very risk averse about change (NBMML Staff).
Need for long term approaches	• I think it’s a ridiculous concept that the government would think they can get outcomes that quickly for people that are in need, and I hate using this term, but this is people, in the too hard basket, these are the most unwell people that we have in our community (Consortium Manager).

#### Understandings of PIR approaches

In the interviews, all respondents provided insights as to their understanding of PIR approaches. Concepts such as recovery and its associated language, consumer directed, coordinated care, and systems change appeared to be well understood and consistent with definitions found in the literature and documents provided by the funding body. However, a lack of understanding by some respondents as to the purpose of PIR was also identified (Table 6).

**Table 6:** Understanding PIR

Subtheme	Representative Quotes
Recovery focus	• Recovery is about identifying the consumer’s needs and where they want to go as far as either maintaining or improving their wellbeing or quality of life (Support Facilitator). • The way their program is built is around recovery, and the language that they use speaks to that. And that’s different from a lot of other community mental health services (Community Support Worker).
Coordination Role	• Cornerstone is the relationships that you have with other organisations and an understanding of what our needs are as an organisation (Consortium Manager). • Coordinated care is that wrap around care for them. That means bringing in the services that they need at the time that they need it (NBMML Staff).
Person centred	• Client driven and individually tailored and that is one of the beauties of the program (Consumer Rep). • That’s embedded in the recovery frame work, they [client] drive – they’re driving (NBMML Staff).
Lack of Understanding	• On ground level, we’ve seen the lack of knowledge and understanding of what Partners in Recovery is about and we educate (Support Facilitator). • A lot of people were confused as to what they actually do and how to contact them and engage their services (Consortium staff). • There have actually been incidences where Partners in Recovery workers appeared to work under a case management model (Health care provider).

## Discussion

The NBMPIR is part of an Australia-wide mental health initiative implemented by 48 separate consortia [26]. The principles underpinning PIR in Australia are the same key organising principles utilised by similar mental health initiatives in other countries [53, 54]. These principles emphasise a model that is client-driven, locally specific, focussed on empowerment [10–13], and that coordinates and integrates the multiple services required to meet client needs [5, 14]. Although this was an early evaluation of the NBMPIR, we found this program had established a consortium and local partnerships which, working together on a recovery approach, was facilitating access to a range of services to meet the needs of an increasing number of local clients with severe and persistent mental illness.

The following discussion is organised according to the aims of the NBMPIR and our evaluation. This includes the establishment of partnerships and governance, improving service coordination and creating systems change. Consideration is given to the facilitators and barriers in implementing NBMPIR and to program evaluation.

### Partnerships and governance

A key objective of NBMPIR related to establishing partnerships and a strong and accountable organisational structure. Two years from receipt of funding, and 1 year since accepting the first client, we found NBMPIR had devoted much time to the governance and management of the Consortium and to establishing a reporting framework. Conflict both within and across organisations is described in the literature as an impediment for effective recovery based service provision [15, 55] that is addressed by clarifying roles and expectations [15]. To this end NBMPIR signed Memoranda of Understanding and Service Level Agreements with all Consortium service provider partners.

### Service coordination

Core to PIR is recovery focussed care facilitation, yet throughout the literature there is widespread inconsistency in the understanding and use of recovery language and practices [15, 16, 56–62]. Nepean Blue Mountains Partners in Recovery ensured that a clearly operationalised recovery focus, aligned strongly with the national PIR framework [36], informed its key documents and guided the implementation of its program. Our informants also confirm that PIR program implementation was consistent with care coordination and recovery support models described in the literature [57, 63, 64]. We identified a range of recovery oriented initiatives in the literature, a number of which were being implemented by NBMPIR. These include peer mentoring which has been reported as beneficial to clients in their recovery journey [65–67]. The NBMPIR have also used peer mentoring of service providers as a means of enhancing the understanding and practice of recovery by co-locating Support Facilitators within partner organisations in an educational and coordinating role. Co-location also helps to integrate care from the bottom up by breaking down barriers between services and clinicians [18].

Clients engaging with PIR are those with severe and persistent mental illness and often present in crisis, requiring immediate services that can be flexibly provided [13, 15]. Nepean Blue Mountains Partners in Recovery established a limited pool of flexible funding to address immediate needs such as crisis accommodation and medications [37, 68]. This allowed NBMPIR to assist clients in need despite client intake at the time of evaluation being at capacity [37, 68].

### Systems change

Systems change is acknowledged as long term [18], however, some evidence of early systemic changes emerged from our evaluation. We noted the increased use of recovery language and practices across the consortium and that siloing of health care providers was being addressed through greater collaboration. We identified improved access to services as a result of enhanced referral pathways and partnerships.

### Facilitators and barriers to implementing PIR in the NBM area

Drawing on the findings of our evaluation including our literature review [37], we identified key facilitators and barriers for NBMPIR. As barriers often result from an absence of facilitators, we considered these together under the following headings related to the key areas our participants described: Alignment of vision and purpose; building an efficient system; getting the message out and sharing information; understanding roles and support and training of staff; building capacity and systems change; addressing service gaps and engaging peers. We consider these findings in relation to the literature, noting areas of common experience and also issues that were specific to NBMPIR. Finally, we reflect on the importance of evaluation.

#### Alignment of vision and purpose

Consortium partners of NBMPIR generally had a similar reform agenda - placing the client at the centre of a recovery based approach to service delivery. Partners also brought specific areas of expertise and unique resources. This enabled the Consortium to address the complex needs of those with severe and persistent mental illness. The importance of such a shared vision and aligned efforts among partner organisations is well recognised [69]. However, barriers to successful collaboration in NBMPIR were observed with some partner approaches to service delivery less well aligned with the PIR model. When there are differences in ideologies, or organisational culture, and no shared philosophy or common goal, the beliefs and attitudes of individuals can adversely impact collaborative service delivery [70].

#### Building an efficient system

There was a marked change in NBMPIR system efficiency when funding was accessed to employ additional Support Facilitators, enabling greater consumer access. System capacity was also expanded by funding support projects and training for partner staff. Interviews further revealed that Support Facilitators supported this training and the development of service partnerships at ground level, an important consideration for integrated recovery-based care [15, 18].

Conversely, many interview and survey respondents commented on bureaucracy and red tape in the early program stages, which adversely impacted decision making. Respondents also commented on procedural guidelines which were unclear and caused confusion. Perceptions of risk with decisions can create avoidance [55, 71], and NBMPIR was observed to be slow in some of its decision making due to perceived risk. In clinical contexts these challenges are reportedly addressed through a shared decision making process which can also enhance efficiency and transparency [71].

Although the NBMPIR Consortium was operating within its means, its effectiveness may have been compromised in the early stages by a substantial amount of unspent funding. The lengthy waitlist after only 2 months of operations suggests that the system could not cope with the recruited client numbers. When funding was released and additional Support Facilitators recruited, the waiting list rapidly reduced.

#### Getting the message out and sharing information

Initial communication and promotion of NBMPIR strongly engaged local communities and was received enthusiastically. A risk noted in our evaluation was the relative lack of subsequent communication particularly with health care providers. They were often quite negative in describing their engagement with NBMPIR which was felt to be inconsistent, with contact and provision of information infrequent. Though the future communications strategy is targeted to specific stakeholders, especially service providers, it will be important to further strengthen and sustain processes for information sharing, as this underpins effective partnerships and integrated health care [70]. It is also important that recovery principles receive continued promotion to sustain the PIR model. Indeed, the Support Facilitators in NBMPIR, like their counterparts in the Australian Capital Territory, were actively taking on this task and this contrasted with other PIR programs in western Sydney where promotion was considered inadequate [22, 31].

#### Understanding roles and supporting and training staff

Most respondents confirmed they understood their role well, although it was also reported that some Support Facilitators were still working in a case management rather than facilitator role. Respondents generally felt that understanding their roles and responsibilities led to effective communication both within the NBMML and across partner organisations. Clarity of individual and organisational roles and responsibilities is an important facilitator of partnerships, enhancing teamwork and job satisfaction [72] with inadequate knowledge or understanding of others’ roles suggested as causing unclear expectations or negative perceptions and non-collaboration [69]. The Co-location project enables Support Facilitators to work in an educational capacity with partner organisations; however, it will be crucial that their roles are clearly defined and understood.

Well-resourced and evidence-based training is important for knowledge transfer and understanding and implementation of recovery practice [12, 73–75]. Survey and interview respondents described challenges including minimal training and a reliance on existing personal skills, though NBMPIR recently finalised an education plan tailored to staff of each stakeholder and provider group.

It is recognised that training must be aligned with the recovery model [63, 76] which can be a challenge in light of the lack of a commonly understood conceptual framework to guide practice [10, 12, 16]. Program documents were aligned with the recovery language guide from the Mental Health Coordinating Council - the peak body for community mental health organisations in New South Wales, and Support Facilitators further modelled recovery based practices [64]. Survey and interview respondents noted a widespread understanding and use of recovery language and recovery-based practices.

#### Building capacity and systems change

The NBMPIR were developing and starting to rollout a number of projects aimed at strengthening recovery approaches and building service capacity in the region. Systems change projects such as co-location will enhance care coordination, whilst smoking cessation and physical fitness programs that include stakeholder training were designed to reduce hospital admissions and mortality [37]. The NBMPIR recognised the importance of the consumer role, and a client/carer regional engagement program was intended to grow a resource of skilled advocates and engage them in leadership roles [37]. The consumer voice at the organisational level is as important as consumer centeredness is at the service level. Indeed, ground level knowledge and information sharing has been identified as crucial [18, 70] and those who have had direct experience relating to mental health can provide powerful insights [15].

#### Addressing service gaps

Clearly, NBMPIR was making a positive difference to the lives of consumers and assisting the effective networking of providers, although, this was a work in progress. Given the short time that PIR had been operating, this program was progressing well.

The NBMPIR were aware of the challenges they face in service delivery especially given the geographical and population diversity of the region. The NBM population has a significant proportion of CALD and Aboriginal and Torres Strait Islander people and these groups have poorer access to mental health care and higher rates of suicidality [77, 78]. Our evaluation at an early stage of PIR did not identify any programs specifically targeting CALD and Indigenous needs. Furthermore, survey and interview respondents indicated a lack of focus on these populations and an absence of CALD and Aboriginal and Torres Strait Islander staff at this time who might facilitate PIR access for these communities. These are important considerations for the NBMPIR but also for similar programs with CALD and Indigenous populations in ensuring that unique needs can be understood and addressed in a culturally appropriate manner. Some groups in the community such as the homeless and those with dual diagnosis were also observed to be missing out on services. Other challenges in access included transport, housing, and social opportunities-the latter was also noted in other PIR programs as an unmet need [32].

#### Engaging peers

Peer support is well evidenced to facilitate recovery approaches in areas of comorbid substance abuse and mental illness and is particularly effective when utilising peers who have been on similar recovery journeys themselves [65–67]. The Wellness Recovery Action Plan (WRAP) is a particularly effective way of utilising peers to help develop consumer strengths [79, 80]. Our interviews suggested that already at this early stage one client engaged with NBMPIR was ready and willing to assist in this way. Should this response be reproduced with other client/carers this will not only help with recovery of others, but will also increase the capacity and efficacy of a very relevant support network.

### Evaluation

Evaluation is often a challenge in implementing a recovery approach [5, 10, 12, 14] including in other PIR programs [32]. Nepean Blue Mountains Partners in Recovery used instruments such as CANSAS and developed databases such as PENELOPE which assist with evaluation. Evaluation activities were embedded within all PIR support programs with quality improvement measured against key performance indicators.

Importantly, the PIR evaluation reported in this paper provided a comprehensive understanding of the early implementation of the NBMPIR. Mapping the program using a PLM was helpful in ensuring all elements of this complex program were considered [81]. Understanding was enhanced through collection of data through document review, surveys and interviews and later thematic analysis. We reported contrasting views and experiences of the program across different participant groups. We often noted that different roles and experiences in NBMPIR elicited different responses to our questions and we could gather more nuanced responses from those participants who answered open-ended survey and in-depth interview questions.

### Limitations

It is important to note that this was an early evaluation of a local program and findings are limited to that context despite their potential relevance for other similar programs. Other programs may have different populations and services that would influence program implementation. Although NBMPIR had developed relationships with a range of health care providers, at the time of evaluation few General Practitioners were engaged with the program. General Practitioners, as key providers of primary health care in the Australian setting, are a source of referral and also important in following up clients engaged with the program. Future evaluations could gain another important perspective on the operations of NBMPIR by collecting information from General Practitioners.

## Conclusions

This study contributes much needed knowledge about the complexities of consumer-centred localised service delivery innovation in mental health care. Although this was an early evaluation of the NBMPIR, we found this program had established a consortium and local partnerships which worked together in a recovery approach to facilitate access to a range of services for an increasing number of clients in the region with severe and persistent mental illness. Support Facilitators were developing new collaborative partnerships with service providers and referral pathways that provided greater access to services. The use of recovery language and practices were becoming more common, especially as these were modelled at the consortium level and promoted by Support Facilitators on the ground. These achievements provide evidence of early systems change.

The PLM approach has proven a useful way of mapping the intended impacts of NBMPIR and the processes and resources required to achieve these. It has also provided a useful framework for this evaluation which provides a baseline for future evaluations conducted by NBMPIR.

The ultimate test for NBMPIR is whether their model of care will make a difference to the lives of consumers. Clearly this is a work in progress, and gaps were identified that need to be addressed, however our evaluation has documented positive changes in the lives of those with severe and persistent mental illness. This has been a remarkable achievement in the 12 months from commencement of PIR services until completion of our data collection.

## Additional files

Additional file 1: Nepean Blue Mountains Partners in Recovery Evaluation Framework (DOCX 111 kb)

Additional file 2: Documents Reviewed (DOCX 16 kb)

Additional file 3: Online survey (DOCX 234 kb)

Additional file 4: Semi structured interview guide (DOCX 207 kb)

Additional file 5: Qualitative coding frame (DOCX 61 kb)

## Data Availability

The data that support the findings of this study are available from Wentworth Healthcare Limited but restrictions apply to the availability of these data, which were used under license for the current study, and so are not publicly available. Data are however available from the authors upon reasonable request and with permission of Wentworth Healthcare Limited.

## Abbreviations

CALD: Culturally and linguistically diverse
CANSAS: Camberwell Assessment of Need Short Appraisal Schedule
Centrelink: Australian Government employment and welfare service
CMIS: Client Management Information System
DBT: Dialectical Behaviour Therapy
FaCS: Family and Community Services
GP: General Practitioner
HASI: Housing and Accommodation Support Initiative
NBMLHD: Nepean Blue Mountains Local Health District
NBMML: Nepean Blue Mountains Medicare Local
NBMPIR: Nepean Blue Mountains Partners in Recovery
NGO: Non-government
PENELOPE: The Client Information Management System (CMIS)
PHaMS: Personal Helper and Mentor Support
PIR: Partners in Recovery
PLM: Program logic model
WRAP: Wellness Recovery Action Plan
