# Supplementary material for: Partners in Recovery: an early phase evaluation of an Australian mental health initiative using program logic and thematic analysis
Source: BMC Health Serv Res. 2019 Jul 26;19:524. doi: 10.1186/s12913-019-4360-2 (PMC6660922; doi:10.1186/s12913-019-4360-2)
Supplement: Supplementary file 1 — Nepean Blue Mountains Partners in Recovery Evaluation Framework (DOCX 111 kb) [file 12913_2019_4360_MOESM1_ESM.docx]

**BMC Additional File 1**

## Additional file 1 - Nepean Blue Mountains Partners in Recovery Evaluation Framework

The Nepean Blue Mountains Partners in Recovery (NBM PIR) Evaluation Framework described in this document is based on a Program Logic Model (PLM) (Kellogg, 2004). The model has been developed by the University of Western Sydney (UWS) research team from a generic model developed in close collaboration with a Project Reference Group convened by the Nepean Blue Mountains Medicare Local (NBMML).

The Inputs, Activities, Outputs, Outcomes and Impacts described in the PLM, as well as the indicators and measures recommended for evaluation, have been aligned throughout this framework to the following relevant documents: NBMML Strategic Plan 2014-2017 referenced by the letters “SP”; the Australian Government DoH - PIR Evaluation Framework (December 2013) referenced as “DoHEF”; and the Australian Government DoH PIR Minimum Client Data Set (MDS) (May 2014) referenced as “MDS”. Consultations with the NBM PIR Regional Manager have further informed the framework.

KEY: **Black font = Original generic framework which includes reference to the NBMML Strategic Plan 2014-17**

**Light Green font = Australian Government DoH - PIR Evaluation Framework (December 2013) (DoHEF)**

**Orange font = Australian Government DoH PIR Minimum Client Data Set (MDS)**

**Dark Blue font = Consultations with NBM PIR Regional Manager**

Other similarly aligned documentation has been colour coded in this Framework and includes: **Establishment Financial Report (EFR)**; **Risk Management Plan (RMP); Establishment Report-Communications (ER-C); 6 monthly Qualitative Performance Report (6QPR); PIR Program Plan 2013-2014 (PIRPP); Annual Activity Work Plan (AAWP); Referrals/demographics Template (RDT); Establishment Performance Report (EPR); PIR Operational Guidelines May 2013 (PIROG).**

**Please note the following stakeholder participants and organisations contributing data in this evaluation framework:**

- - Clients
  - Carers
  - Community representatives
- Health care providers
  - Psychologist
  - Counsellor
  - Psychiatrist
  - General Practitioner
  - Nurse
  - Allied health
  - Other (TBA)
- Board/ Management/Staff of one of the consorting organisations/partners/agencies
  - PIR Lead Organisation (includes support facilitators)
  - NBM Local Health District
  - Community Clinical Mental Health Team
  - Personal Helper and Mentors (PHaMS)
  - Housing and Accommodation Support Initiative (HASI)
  - Day program provider
  - Sustenance and short term shelter provider
  - Housing
  - Employment
  - Drug and Alcohol

o Disability services o Legal Services

- - Other (TBA)

| **1. Inputs** include the human, financial, organizational, and community resources a program has available to direct toward doing the work. | | | | |
| --- | --- | --- | --- | --- |
| **Performance Indicators** | **Survey** | **Interview** | **Document Review** | **Document/Measure** |
| **1.1 Funding** | | | | |
| - Sources of funding (including flexible funding for “crisis” and immediate access – also DoHEF pp. 16-17) - Funding allocated (and timed) according to program priorities. - Amount and adequacy of funding allocated/dispersed |  | x  x | x | As recorded in PIRPP (p.4) (AAWP p. 2)  Documented budget dispersal and acquittal (EFR) 6QPR (p.7) (AAWP p.2 )  - includes proportion for “prevention” focus |
| **1.2 Management/governance structures (DoHEF p.21)** | | | | |
| NBM PIR demonstrates a “robust culture” including openness and transparent processes and reporting accountability   - Clinical and corporate governance protocols established (PIRPP   p.2)   - Program Finances Audited - Risk Management Plan developed and used - Organisational and management support for the program - Client Information Management Systems (CIMS) in place (MDS p.9) PIRPP (p.8) AAWP (p.6) - Operational “reporting framework” to DoH in place (DoHEF p.20) PIRPP (p.8); PIROG (pp. 26-31) | x | x | x | Clinical governance framework  documented (EPR 6)  Audit reports  Documented risk assessment (uses risk management plan [RMP] for reporting) NBMML management support (monitoring and reporting to DoH requirements PIRPP p.8)  Collection and reporting of Minimum Client Data Set (MDS) (DoHEF 1.3.2; MDS 1.4 p.3)  Consultation with DoH and alignment of information provided by PIR Org against framework. |
| **1.3 Key Stakeholder Staff - PIR Organisation/partners/agencies/NGO service providers** | | | | |
| Staffing according to program requirements |  |  | x | Program specific expertise identified and sourced (EFR –“recruitment and training”). |

| Clear staff job descriptions aligned to work contracts | x |  | x | Staff PDs  Staff satisfaction with job descriptions |
| --- | --- | --- | --- | --- |
| Engagement of Key stakeholders |  |  | x | Consortium Stakeholder numbers, skills,  input to PIR program (also GPs, Allied Health, Other) (SP3.1.1, 3.1.2.) Formalised partnerships (EPR p. 4) MoU and management committees finalised –PIRPP (pp.1-3) EPR (p.3). Service level agreements in place (EPR p.3) |
| Defined roles of Stakeholders including organisations and groups |  |  | x | Terms of reference established  Mechanisms in place for reporting stakeholder activity to Lead Organisation (PIRPP-p.2) |
| **1.4 Community/consumer stakeholders** | | | | |
| Community organisations engaged as part of PIR Consortium |  |  | x | Number and range of organisations. |
| Consumers participating in program (SP4.2.2) (AAWP p.6) |  |  | x | Register of consumer reps on PIR  program related committees  Program reported to consumer forums |
| Consumer satisfaction with interactions including consultation and  opportunities for feedback to improve PIR program | x | x |  | Consumer satisfaction with consultations |
| **1.5 Research/evaluation expertise** | | | | |
| Identified research/ evaluation goals/priorities |  |  | x | Evaluation framework described in Program plan |
| Agreements - research partners/ documented internal processes (SP6.1) (EPR p.6) |  |  | x | Contracts engaged/funded (EFR)  Evaluation/research plan documented  and approved (SP6.1.1) PIRPP (p.8) |

| **1.6 Technology including Information Technology (DoHEF p.21)** | | | | |
| --- | --- | --- | --- | --- |
| Adequate IT resources and support for the program |  |  | x | Description including numbers and  expenditure (Also “asset register” in Establishment Financial Report). Documented evidence of resource acquisition  Web-hosted data base-restricted access to PIR partnership staff (not for DoH) |
| Staff and stakeholder satisfaction with allocation per person and efficacy | x | x |  | Satisfaction and efficacy reported via  survey and interview |
| **1.7 Other resources that are program specific** | | | | |
| Transport: car leased by NBM PIR |  |  | x | Leasing contract, running and  maintenance costs recorded |

**2. Activities** refer to the way resources are utilised. Activities are the processes, tools, events, technology, and actions that are an intentional part of the program implementation. These interventions are used to bring about the intended program changes or results.

| **Indicators** | **Survey** | **Interview** | **Document Review** | **Document/Measure** |
| --- | --- | --- | --- | --- |
| **2.1 Program planning and development** | | | | |
| Establish project reference group with “key” personnel to feed back to consortium (e.g. Consortium Advisory group –“CAG”) |  |  | x | Schedule of meetings, corresponding minutes and action items (EPR p. 4). |
| Establish a “Framework of Language” understood across consortium | x | x | x | Documented framework, and survey and interview reports understandings. |

| Consultations by NBM PIR with service provider stakeholders, as well as consumers and researchers to inform “local” program development and implementation (SP1.1.2, 3.2.1) (PIRPP p.10). | x |  | x | Documentation of consultations and  forums  Stakeholders profiled and mapped for appropriate involvement (Establishment Report - Communications [ER-C] template-pp.2, 10) (PIRPP- p.9). Documented program plan - alignment with Service Coordination Manual (EPR pp. 7-8) |
| --- | --- | --- | --- | --- |
| Develop agenda of “subprograms” through various consortium partners focused on *a shared vision of systems change* (e.g. housing, legal, transport, Centrelink…) (See AAWP p.8) |  | x | x | Program plan notes input/ consultations  and forums-prioritisation (AAWP) Minutes of meetings, central review document (ER-C p.3)  Description of subprograms and how they would impact on systems change |
| Service provider stakeholder and consumer satisfaction with input to local program planning and development | x | x |  | Satisfaction reported via survey and interview |
| Program is informed by evidence |  | x | x | Program plan notes evidence base Evaluation framework established |
| Alignment with other stakeholder Strategic Plans |  | x | x | Program plan notes alignment with  other stakeholder strategic plans including shared strategic plan e.g. with LHD. (SP3.1.2) |
| Communication/ media strategies developed to inform relevant stakeholders about program (SP1.1.6) PIRPP (p.9) |  |  | x | Publicity noted in Program Plan ER-C  p.11  Comms via ER-C template 1.1.6 pp. 5-7 |
| **2.2 Client needs identification (DoHEF p.20)** | | | | |
| Develop/establish “assessment intake tool” to enable identification of client needs and recovery plan (DoHEF pp. 24-25) (PIRPP p.6). |  | x | x | Tool measures against five  predetermined intake data areas (MDS pp.6-9) |

| Develop tool to assess client “progress” |  |  | x | Data extracted through MDS and CIMS  (PIRPP p.6 “exit framework”) |
| --- | --- | --- | --- | --- |
| Develop contextualised “care coordination and support model” (DoHEF p.19) |  | x | x | Documentation of development  processes e.g. workshop/consultation reports  Range and type of model (e.g. adaptive recovery-focused AAWP p.3) |
| **2.3 Stakeholder education and support** | | | | |
| Develop an education plan for providers to understand “recovery” |  |  | x | Documentation of education plan. PIRPP  (p.5)  Recovery measured multifactorially not only on “28 day hospital readmission” criteria |
| Develop program to train support facilitators in-line with “local” conditions |  |  | x | Documented program plan |
| Establish a facilitators support working group |  |  | x | Meetings, minutes, action items |
| PIR Lead Organisation staff support (to meet client crisis needs) until relevant partner appoints facilitator staff | x | x |  | Lead organisation staff satisfaction with support reported via survey and interview |
| Stakeholder and staff satisfaction with education/support | x | x |  | Stakeholder and staff satisfaction with  education and support reported via survey and interview |

| **3. Outputs** are the direct products of activities and may include new resources and/or types, levels and targets of services and programs delivered by the NBMML. | | | | |
| --- | --- | --- | --- | --- |
| **Indicators** | **Survey** | **Interview** | **Document Review** | **Document/Measure** |
| **3.1 Program Implemented** | | | | |
| Staff recruited, oriented, supported to implement program (DoHEF  p.18) |  |  | x | Program report, Annual report Staffing plans/occupancy  See also 6QPR (p.2); PIRPP (pp.5,12,13) |
| Staff satisfaction with orientation and support | x | x |  | Reported satisfaction via survey and interview |
| Program implemented consistent with “care coordination and  support model” (DoHEF p.19, 24) |  |  | x | Program report including attendance, delivery according to plan, participant satisfaction See also 6QPR (p.2) |
| Level of client intake is in line with Program expectations (DoHEF p.  12) Effective intake referral pathways developed (PIRPP p.6) |  | x | x | Number and proportion of clients  referred by source (see RDT)  MDS data  6QPR (p.2) |
| Client “progress” measurement is established |  |  | x | Document review (e.g. MDS, CIMS data) |
| Consumer, Consortium and other service provider stakeholders engaged in program implementation |  |  | x | Program reports (also ER-C 1.1.6 p.5,  and ongoing stakeholder mapping and profiling p.10)  Documented engagement via criteria ER-C p.12  6QPR (p.2) |
| Consumers, Consortium and other service provider stakeholder  satisfaction with engagement in program (DoHEF p. 24) | x | x |  | Consumers, Consortium and other service provider stakeholders reported satisfaction via surveys and interviews |
| Lead agency and partner organisations operate effectively as a consortium in the establishment and early implementation phases | x | x |  | Online survey of PIR (partnership  assessment tool)  Face to face and telephone |

| (DoHEF p. 23) |  |  |  | consultations with “local” PIR  organisations as available from national data collection (DoHEF p.23) 6QPR (p.3) |
| --- | --- | --- | --- | --- |
| PIR organisation is operating in an efficient and cost effective manner (DoHEF p.17) | x | x | x | Program costs (establishment and  recurrent - see EFR)  Reported level of effectiveness and efficiency (client loads and outcomes) 6QPR (p.2) |
| Publicity strategies implemented (SP1.1.6) (ER-C pp. 5-8; p.11) |  |  | x | Media releases distributed to local media PIRPP (p. 9)  Website and other communications |
| **3.2 Evaluation of program** | | | | |
| Expertise engaged as required |  |  | x | Research and other contracts (SP6.1.1) |
| Active participation in “local” monitoring and evaluation |  | x | x | Client, and systems level data provided in line with reporting framework -  6QPR (p.2) |
| Evaluation informs future program development and innovation (SP 6.1.4, 6.1.3) | x | x | x | Documentation of evaluation and  planning, and use of feedback  (ER-C p. 8, p.12 “act, review, improve”) |
| Consortium and other service provider Stakeholder staff/consumer experience of evaluation focus | x | x |  | Consortium and other service provider  Stakeholder staff/consumer reporting of evaluation focus in survey and interviews |

| **4. Outcomes** are the specific changes in program participants’ behaviour, knowledge, skills, status and level of functioning. | | | | |
| --- | --- | --- | --- | --- |
| **Indicators** | **Survey** | **Interview** | **Document Review** | **Document/Measure** |
| **4.1 Consumers and all other stakeholders and providers are informed about program** | | | | |
| Consumers seek information on program |  |  | x | Website visits, phone enquiries,  turnover of printed material, consultation. Tally kept at point of referral on source of information. |
| Service provider staff and other relevant Stakeholder awareness of program | x | x |  | Service provider staff and other  relevant Stakeholder reported  awareness in survey and interviews |
| **4.2 Improved consumer access to health program (as relevant)** | | | | |
| Consumers access PIR services | x | x | x | Reports on numbers, types and  frequency of services accessed (e.g. transport etc.)  Number and type of onward referrals to other agencies  Satisfaction |
| **4.3 Program achieves stated outcomes** | | | | |
| People with severe, persistent mental illness and complex needs are  supported through PIR as an effective coordination model (DoHEF p. 11) |  | x |  | Face to face consultations with key PIR stakeholders Also 6QPR (p.4) |
| Improvement in consumer health status and level of functioning (DoHEF pp. 10-11) - includes reduced burden and increased well- being for carers | x | x |  | Carer assessment tool (DoHEF p.11)  Client self-report (quality of life) Client and carer satisfaction surveys Face to face consultations with PIR clients and carers/family (DoHEF p11)  NMDS data- hospital admissions and  contact with community mental health as per national data collection |

| Improvement in provider and staff knowledge, skills, level of functioning | x | x |  | Reported improvement through survey and interviews |
| --- | --- | --- | --- | --- |
| Service providers engage in new and more effective partnerships to meet needs of people with severe and persistent mental illness and complex needs (DoHEF pp. 13-14) | x | x | x | Number/types of partnerships  Improved partnership score (partnership assessment tool) as per national data collection |

**5. Impacts** are the fundamental intended or unintended change occurring in organizations, communities or systems as a result of program activities.

| **Indicators** | **Survey** | **Interview** | **Document Review** | **Documents/Measures** |
| --- | --- | --- | --- | --- |
| **5.1 Enhanced local community health and wellbeing (AAWP p.9)** | | | | |
| PIR clients and their family/carers have improved access to required and culturally appropriate services and supports (DoHEF p.16) | x | x | x | Reported improvement in services  access as per national data collection  see also 6QPR (p.4, p.5)  ED Hospital presentations and admissions for mental illness |
| Sustained improvement in health outcomes where PIR program has contributed, including changes in morbidity and mortality and in prevalence of risk factors in the local community |  |  | x | As relevant - comparison of local  program specific pre and post implementation data including national data such as. suicide rates (MoH Data, My Healthy Communities) |
| Increased health equity where PIR program has contributed |  |  | x | Program addresses community health  gap (SP1.1) (MoH Data, My Healthy Communities related to mental health) |
| **5.2 Integrated and coordinated health services (SP3.2.2 and AAWP** **p.9)** | | | | |
| Program contribution to shared engagement between all stakeholder partners | x | x | x | Documentation of shared engagement  and reporting of same via survey and interviews e.g. ER-C 1.1.7 p.10, p.12 |

| Level of coordination between clinical and community support service providers is improved DoHEF p. 12) | x | x |  | Partnership assessment tool as per national data collection  6QPR (p.3) |
| --- | --- | --- | --- | --- |
| PIR Clients have improved access to stable housing and increased participation in employment, education & social activities (DoHEF p.10) | x | x |  | PIR MDS - CANSAS outcomes/self- report (MDS p.4) |
| Clinical and community support services are operating according to a community-based “recovery” model (DoHEF p. 14) | x | x |  | Reported examples of service system  “reform” influenced by PIR 6QPR (pp.5- 7)  Level of shared understanding reported |
