# Supplementary material for: Partners in Recovery: an early phase evaluation of an Australian mental health initiative using program logic and thematic analysis
Source: BMC Health Serv Res. 2019 Jul 26;19:524. doi: 10.1186/s12913-019-4360-2 (PMC6660922; doi:10.1186/s12913-019-4360-2)
Supplement: Supplementary file 2 — Documents Reviewed (DOCX 16 kb) [file 12913_2019_4360_MOESM2_ESM.docx]

**BMC Additional File 2**

## Additional file 2 - Documents Reviewed

| 1. Australian Government Guidance Documents |
| --- |
| Australian Government Department of Health (DoH) - PIR Evaluation Framework   1. DoHEF (December 2013) |
| Australian Government Department of Health - PIR Minimum Client Data Set - Version 1.3   1. MDS (May 2014) |
| Australian Government Department of Health and Aging: Partners in Recovery Operational Guidelines (Annexure B)   1. PIROG (May 2013) |
| 1. **Documents for DoH Reporting** |
| NBM Partners in Recovery Program Plan 2013-14   1. PIRPP (September 2013-June 2014) |
| Partners in Recovery Client Activity Reports   1. CAR-1 (Referrals and demographics [RDT] to 31 March 2014) 2. CAR-2 (To 30 June 2014) 3. CAR-3 (to 30 September 2014) |
| Establishment Performance Reports   1. EPR (one off performance report for 2013-2014) 2. ER-C (one off communications report 2013-2014) 3. RMP (one off risk management plan 2013-2014)   6 Monthly Performance Reports (Qualitative Reporting)   1. 6QPR (Establishment template) 2. 6QPR-2 (September 2013 to July 2014) |
| 6 Monthly Performance Reports- “Expenditure”   1. EFR (Establishment 2012-2013) 2. 6PRE-1 (September 2014) |
| Annual Activity Work Plan 2014-2015   1. AAWP-1 (draft) 2. AAWP-2 (final) |
| 1. **Nepean Blue Mountains (PIR) – Internal Documents** |
| NBM Partners in Recovery Mapping Report (Just Health Consultants)   1. (September 2013) |
| NBM Partners in Recovery Program Manual (V.01)   1. Establishment (September 2013)   b.1 V.1.1 (March 2014) |
| Audit Reports   1. September 2014 |
| Support Facilitator Working Group Monthly Minutes   1. February 2014 2. March 2014 3. April 2014 4. May 2014 5. June 2014 6. July 2014 7. August 2014 8. September 2014 9. October 2014 |
| PIR Consortium Meetings - Minutes  m.1 July 2014  m.2 August 2014  m.3 September 2014  m.4 October 2014  m.5 November 2014  m.6 December 2014 |
| Memorandum of Understanding   1. MoU (June 2014) |
| Service Level Agreements   1. SLA (2014) 2. SLA-R (RichmondPRA) 3. SLA-V (variation for UCMH- August 2014) |
| NBM PIR Service Coordination Manual   1. SCM (draft) |
| Education Plan   1. EP (Final) |
| Final Deed- Contract DoH and NBMML   1. Schedule 38 |
| 1. **Other Documents** |
| Regional Forum Notes (November 2012)   1. Blue Mountains 2. Nepean 3. Windsor 4. Lithgow |
| Guidance notes   1. Consumer transfers, suspensions and exits |
| Project and research proposals   1. Systems change (July 2014) |
| NBM PIR Recovery Model   1. Tender for Recovery Model |
| NBM Organisational hierarchy   1. SC- (Staff Chart) |
| NBM PIR Consumer and Referrer Information   1. Information pack and pamphlets |
| NBM PIR Partnerships Analysis Tool (from Victorian Health)   1. PAT - Partnerships Analysis Tool |
| NBM PIR Media Releases   1. Newsletter December 2013 2. Newsletter March 2014 3. Newsletter September 2014 4. Media release December 2014 |
| PIR Assessment Intake Tool   1. CANSAS |
| Mental Health Coordinating Council (MHCC)   1. MHCC Recovery Language Guide |
