# Supplementary material for: Partners in Recovery: an early phase evaluation of an Australian mental health initiative using program logic and thematic analysis
Source: BMC Health Serv Res. 2019 Jul 26;19:524. doi: 10.1186/s12913-019-4360-2 (PMC6660922; doi:10.1186/s12913-019-4360-2)
Supplement: Supplementary file 3 — Online survey (DOCX 234 kb) [file 12913_2019_4360_MOESM3_ESM.docx]

## Additional file 3 - Online survey


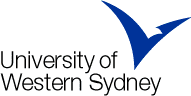


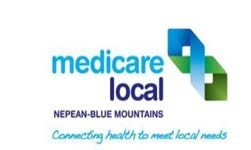


**On-line Survey**

**Nepean Blue Mountains Partners in Recovery**

**(NBM PIR) Evaluation**

**Survey items (n=45) correspond with the following relevant PLM components:**

**Inputs = questions 1-4 (PLM 1.2, 1.3, 1.4, 1.6)**

**Activities = questions 5-8 (PLM 2.1, 2.3)**

**Outputs = questions 6, 8-11 (PLM 3.1, 3.2)**

**Outcomes = questions 12-14 (PLM 4.1, 4.2, 4.3)**

**Impacts = questions 15-16 (PLM 5.1, 5.2)**

*The following survey is designed to gain information on your interaction with the Partners in Recovery (PIR) program in the Nepean Blue Mountains area. This information will be used to inform the ongoing operations of the program*

*Completing this survey will be taken as your consent for participation.*

**The following survey will take approximately *15* minutes to complete**

We are seeking information related to your “primary” role with Partners in Recovery (PIR). If you wish to also respond in another role please indicate at the end of the survey with your contact details and the Research Officer will provide an additional survey for you in respect to that role.

**At the end of the survey you can tell us if you would like to also take part in an interview. If you provide your contact details the research officer will contact you to make suitable arrangements.**

**Please provide one response for each of the following**

I am: Female Male

My age group is:

18-30

31-50

> 51

My primary involvement with Partners in Recovery in the Nepean Blue Mountains area is as a: Consumer

Carer

Community representative

Board/ Management/Staff of one of the consorting organisations/partners/agencies

Which stakeholder organisation?

PIR Lead Organisation (including support facilitator) Local Health District

Community Clinical Mental Health Team

Personal Helper and Mentors (PHaMS)

Housing and Accommodation Support Initiative (HASI) Day program provider

Sustenance and short term shelter provider

Housing

Employment

Drug and Alcohol Disability services Legal Services

Other (please specify)……………………………………………………………………………………………….

What is your role?

Board

Management

Staff member Health care provider: Psychologist

Counsellor

Psychiatrist

General Practitioner

Nurse

Allied health

Other role not included above (please specify) …………………………………………………………………

In what capacity are you responding (please tick one)?

As an individual

As a representative of your organisation or discipline

If you wish to respond in both capacities, please complete details at the end of the survey so an additional survey can be provided to you

You will now be directed through the survey according to the information you provided above.

**1.** *Consortium and other service provider stakeholder Board, staff and management*

The Partners in Recovery Program seeks to create a strong culture with good management and governance structures.

Please tick the box that best matches your response to the following statements:

| The organisation and management  of Partners in Recovery is open and transparent including in its reporting | Strongly  disagree | Disagree | Neutral | Agree | Strongly  agree | Don’t  know |
| --- | --- | --- | --- | --- | --- | --- |

| The management and oversight  structures for Partners in Recovery enables effective and efficient management | Strongly  disagree | Disagree | Neutral | Agree | Strongly  agree | Don’t  know |
| --- | --- | --- | --- | --- | --- | --- |

Could you comment further on the management and governance practices of PIR?

………………………………………………………………………………………………………………………………………………………

……………………………………………………………………………………………………………………………………………………..

**2.** *PIR Consortium staff and management*

Partners in Recovery seek to draw on expertise from across its region. The staff working on

Partners in Recovery are crucial for the delivery of PIR services.

Please tick the box that best matches your response to the following statements:

| I have a clear job  description | Strongly  disagree | Disagree | Neutral | Agree | Strongly  agree | Don’t  know |
| --- | --- | --- | --- | --- | --- | --- |

| My daily work practices  match my defined role | Strongly  disagree | Disagree | Neutral | Agree | Strongly  agree | Don’t  know |
| --- | --- | --- | --- | --- | --- | --- |

**3.** *Consumer/community group reps, consumers and carers*

Partners in Recovery seek to engage consumers and community members in all its work. Please tick the box that best matches your response to the following statements:

| Consumers are consulted by  Partners in Recovery | Strongly  disagree | Disagree | Neutral | Agree | Strongly  agree | Don’t  know |
| --- | --- | --- | --- | --- | --- | --- |

| Consumers have enough opportunity  to provide feedback in order to improve Partners in Recovery in the Nepean Blue Mountains area | Strongly  disagree | Disagree | Neutral | Agree | Strongly  agree | Don’t  know |
| --- | --- | --- | --- | --- | --- | --- |

Could you comment further on your interaction with Partners in Recovery?

…………………………………………………………………………………………………………………………………………………………

……………………………………………………………………………………………………………………………………………………………

**4.** *PIR Consortium management and staff*

The use of technology including information technology underpins the efficient operations of the

Partners in Recovery.

Please tick the box that best matches your response to the following statements:

| The IT resources provided by the  Partners in Recovery are sufficient for my requirements | Strongly  disagree | Disagree | Neutral | Agree | Strongly  agree | Don’t  know |
| --- | --- | --- | --- | --- | --- | --- |

| The IT resources provided by the  Partners in Recovery assist to improve communication | Strongly  disagree | Disagree | Neutral | Agree | Strongly  agree | Don’t  know |
| --- | --- | --- | --- | --- | --- | --- |

| The available training for IT use is  adequate | Strongly  disagree | Disagree | Neutral | Agree | Strongly  agree | Don’t  know |
| --- | --- | --- | --- | --- | --- | --- |

| I am efficient in my use of IT  resources in my work with  Partners in Recovery | Strongly  disagree | Disagree | Neutral | Agree | Strongly  agree | Don’t  know |
| --- | --- | --- | --- | --- | --- | --- |

**5.** *Community group reps, Consortium and other service provider staff and management*

Partners in Recovery seek to establish a shared understanding of the language used across the consortium.

Please tick the box that best matches your response to the following statements:

| Partners in Recovery has engaged  my input in establishing a  “framework of language” | Strongly  disagree | Disagree | Neutral | Agree | Strongly  agree | Don’t  know |
| --- | --- | --- | --- | --- | --- | --- |

| A framework of language assists in  building an understanding of the purpose of PIR | Strongly  disagree | Disagree | Neutral | Agree | Strongly  agree | Don’t  know |
| --- | --- | --- | --- | --- | --- | --- |

| The language is easily understood  and used consistently across the consortium | Strongly  disagree | Disagree | Neutral | Agree | Strongly  agree | Don’t  know |
| --- | --- | --- | --- | --- | --- | --- |

| I am satisfied with how the  framework of language is contributing to the efficiency of PIR | Strongly  disagree | Disagree | Neutral | Agree | Strongly  agree | Don’t  know |
| --- | --- | --- | --- | --- | --- | --- |

Can you please explain your response?

………………………………………………………………………………………………………………………………………………………

………………………………………………………………………………………………………………………………………………………

**6.** *Consumers/community reps, consumers and carers, service providers and all other stakeholders* Partners in Recovery seek to work closely with consumers and other stakeholders to develop, set up and run the program in the Nepean Blue Mountains area.

Please tick the box that best matches your response to the following statements:

| Partners in Recovery actively  engages my organisation/ discipline in planning their programs | Strongly  disagree | Disagree | Neutral | Agree | Strongly  agree | Don’t  know |
| --- | --- | --- | --- | --- | --- | --- |

| Partners in Recover actively  engages my organisation/ discipline in running Partners in Recovery | Strongly  disagree | Disagree | Neutral | Agree | Strongly  agree | Don’t  know |
| --- | --- | --- | --- | --- | --- | --- |

| I am satisfied with my level of  input to the local Partners in  Recovery program | Strongly  disagree | Disagree | Neutral | Agree | Strongly  agree | Don’t  know |
| --- | --- | --- | --- | --- | --- | --- |

Can you please explain your response?

……………………………………………………………………………………………………………………………………………………..

**7.** *Lead organisation management and staff*

There are times when “crisis” needs of consumers must be met by PIR Lead Organisation staff until facilitator staff can be appointed.

Please tick the box that best matches your response to the following statement:

| Partners in Recovery provide me  with adequate support to assist consumers in crisis. | Strongly  disagree | Disagree | Neutral | Agree | Strongly  agree | Don’t  know |
| --- | --- | --- | --- | --- | --- | --- |

**8.** *Consortium and other service provider staff and management*

It is important that education and support are provided to all Partners in Recovery partners and staff.

Please tick the box that best matches your response to the following statement:

| I am satisfied with the level of  education and support provided to me for my role in Partners in Recovery | Strongly  disagree | Disagree | Neutral | Agree | Strongly  agree | Don’t  know |
| --- | --- | --- | --- | --- | --- | --- |

Can you please explain your response?

……………………………………………………………………………………………………………………………………………………………

**9.** *Consortium Boards, management and staff*

Partners in Recovery rely on effective working relationships between all the organisations engaged in this work.

Please tick the box that best matches your response to the following statement:

| The Partners in Recovery  consortium is working together effectively | Strongly  disagree | Disagree | Neutral | Agree | Strongly  agree | Don’t  know |
| --- | --- | --- | --- | --- | --- | --- |

Can you please explain your response?

……………………………………………………………………………………………………………………………………………………………

……………………………………………………………………………………………………………………………………………………………

**10.** *Lead Organisation Board, management and staff*

The efficiency of Partners in Recovery is also reliant on being cost effective. Please tick the box that best matches your response to the following statement:

| Partners in Recovery is operating  in an effective and cost efficient manner | Strongly  disagree | Disagree | Neutral | Agree | Strongly  agree | Don’t  know |
| --- | --- | --- | --- | --- | --- | --- |

**11.** *All participants*

Evaluation is crucial to understand what is working and what is not.

Please tick the box that best matches your response to the following statements:

| Partners in Recovery has a strong  focus on evaluating its program  *[All participants]* | Strongly  disagree | Disagree | Neutral | Agree | Strongly  agree | Don’t  know |
| --- | --- | --- | --- | --- | --- | --- |

| Partners in Recovery consults my  organisation/ discipline for feedback on the PIR program *[consumer rep, health and other service provider stakeholder]* | Strongly  disagree | Disagree | Neutral | Agree | Strongly  agree | Don’t  know |
| --- | --- | --- | --- | --- | --- | --- |

| The feedback from the evaluation is  used to inform further Partners in Recovery work *[Lead Org board, management and staff]* | Strongly  disagree | Disagree | Neutral | Agree | Strongly  agree | Don’t  know |
| --- | --- | --- | --- | --- | --- | --- |

Could you comment further on the evaluation focus in Partners in Recovery (all participants)?

………………………………………………………………………………………………………………………………………………………

………………………………………………………………………………………………………………………………………………………

**12.** *Consumer reps, consumers and carers, all service providers, other stakeholder staff and management* Partners in Recovery seek to work closely with all stakeholders to increase their knowledge and awareness of their programs.

Please tick the box that best matches your response to the following statements:

| I have good awareness of Partners in  Recovery programs *[consumer reps, consumers and carers, service providers, other stakeholder staff and management]* | Strongly  disagree | Disagree | Neutral | Agree | Strongly  agree | Don’t  know |
| --- | --- | --- | --- | --- | --- | --- |

| My knowledge of Partners in Recovery  enables me to work effectively as a service provider *[health care and other provider stakeholder staff and management]* | Strongly  disagree | Disagree | Neutral | Agree | Strongly  agree | Don’t  know |
| --- | --- | --- | --- | --- | --- | --- |

**13.** *Consumer and carer, health and other provider stakeholder staff, board and management*

One aim of providing information across the local community is to increase consumer access to

Partners in Recovery services.

Please tick the box that best matches your response to the following statements:

| As a consumer, Partners in Recovery  has assisted me in getting the right services for me *[Consumers and carers]* | Strongly  disagree | Disagree | Neutral | Agree | Strongly  agree | Don’t  know |
| --- | --- | --- | --- | --- | --- | --- |

| As a service provider I am able to  access consumer services through Partners in Recovery *[health and other provider stakeholder staff]* | Strongly  disagree | Disagree | Neutral | Agree | Strongly  agree | Don’t  know |
| --- | --- | --- | --- | --- | --- | --- |

| As a member of Partners in Recovery  staff I am able to coordinate services for Partners in Recovery consumers *[Lead Org board, management, staff]* | Strongly  disagree | Disagree | Neutral | Agree | Strongly  agree | Don’t  know |
| --- | --- | --- | --- | --- | --- | --- |

What else could help increase the level of consumer access to PIR services *[listed participants]*?

………………………………………………………………………………………………………………………………………………………

………………………………………………………………………………………………………………………………………………………

**14.** *Consumers and carers, health and other service provider stakeholder staff*

Partners in Recovery seek to improve the functioning of all those involved.

Please tick the box that best matches your response to the following statements:

| My health and ability to function has  improved through my engagement  with Partners in Recovery *[consumers and carers]* | Strongly  disagree | Disagree | Neutral | Agree | Strongly  agree | Don’t  know |
| --- | --- | --- | --- | --- | --- | --- |

| I have greater hope for my future health and functioning because of Partners in Recovery  *[consumers and carers]* | Strongly  disagree | Disagree | Neutral | Agree | Strongly  agree | Don’t  know |
| --- | --- | --- | --- | --- | --- | --- |

| As a result of my engagement with  Partners in Recovery, my skills and level of functioning have improved *[health and other service provider stakeholder staff]* | Strongly  disagree | Disagree | Neutral | Agree | Strongly  agree | Don’t  know |
| --- | --- | --- | --- | --- | --- | --- |

| PIR has assisted me to engage in new  and effective partnerships that help me meet the needs of my consumers *[health and other service provider stakeholder staff]* | Strongly  disagree | Disagree | Neutral | Agree | Strongly  agree | Don’t  know |
| --- | --- | --- | --- | --- | --- | --- |

Could you comment further on this please?

…………………………………………………………………………………………………………………………………………………………...

……………………………………………………………………………………………………………………………………………………………

**15.** *All participants*

PIR aims to leave a lasting legacy of improved health and wellbeing in its local community. Please tick the box that best matches your response to the following statements:

| Partners in Recovery has resulted in  sustained improvement in consumer access to required services and supports *[Consortium board, management and Staff, health and other providers, Consumer reps, consumers and carers]* | Strongly  disagree | Disagree | Neutral | Agree | Strongly  agree | Don’t  know |
| --- | --- | --- | --- | --- | --- | --- |

| Partners in Recovery has established  improved referral pathways for consumers of CALD and Indigenous background *[Consortium board, management and staff, health and other provider staff]* | Strongly  disagree | Disagree | Neutral | Agree | Strongly  agree | Don’t  know |
| --- | --- | --- | --- | --- | --- | --- |

Could you comment further on this please?

……………………………………………………….……………………………………………………………………………………………………

…………………………………………………………………………………………………………………………………………………………….

**16.** *All participants*

PIR aims to achieve large scale “systems change” through integrated and coordinated health services.

Please tick the box that best matches your response to the following statements:

| Partners in Recovery has assisted my  ability to network with other stakeholders in responding to consumer needs *[Consortium Board, management and Staff, health and other service provider staff and management]* | Strongly  disagree | Disagree | Neutral | Agree | Strongly  agree | Don’t  know |
| --- | --- | --- | --- | --- | --- | --- |

| Partners in Recovery has improved consumer access to integrated services that address multiple needs *[Consumer reps, consumers and carers, Consortium Board, management, staff]* | Strongly  disagree | Disagree | Neutral | Agree | Strongly  agree | Don’t  know |
| --- | --- | --- | --- | --- | --- | --- |

| Clinical and community support  providers are working better together as a result of Partners in Recovery *[health and other service provider staff]* | Strongly  disagree | Disagree | Neutral | Agree | Strongly  agree | Don’t  know |
| --- | --- | --- | --- | --- | --- | --- |

| My organisation/ discipline has a  good understanding of the  “recovery” model of care *[health and other service provider staff]* | Strongly  disagree | Disagree | Neutral | Agree | Strongly  agree | Don’t  know |
| --- | --- | --- | --- | --- | --- | --- |

What systems change have you noticed as a result of Partners in Recovery *[all participants]*?

……………………………………………………………………………………………………………………………………………………………

**Final Question *(all participants)*:**

If there was one message you would like to pass on to Partners in Recovery that would further assist them in improving their programs, it would be ………….……………………………………………………………………..

……………………………………………………………………………………………………………………………………………………..

…………………………………………………………………………………………………………………………………………………….

***Partners in Recovery and the researchers from UWS who are conducting this survey on their behalf, thank you for your participation***

***Do you wish to complete an additional survey in relation to another role you have with Partners in recovery? YES NO***

***Are you also willing to participate in an interview? YES NO***

***If yes for any of the above, please provide your preferred contact details and our Research Officer will contact you to explain the processes involved and make arrangements with you.***

***Name: …………………………………………………………………………………………………. Daytime telephone: …………………………………………………………………………….. Email: ………………………………………………………………………………………………….. Other: ……………………………………………………………………………………………***
