# Supplementary material for: Partners in Recovery: an early phase evaluation of an Australian mental health initiative using program logic and thematic analysis
Source: BMC Health Serv Res. 2019 Jul 26;19:524. doi: 10.1186/s12913-019-4360-2 (PMC6660922; doi:10.1186/s12913-019-4360-2)
Supplement: Supplementary file 4 — Semi structured interview guide (DOCX 207 kb) [file 12913_2019_4360_MOESM4_ESM.docx]

## Additional file 4 – Semi structured interview guide


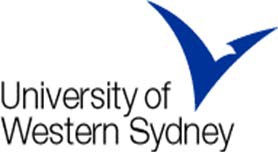


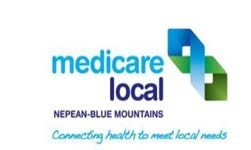


**Semi-structured Interview Guide**

**Nepean Blue Mountains Partners in Recovery (NBM PIR) Evaluation**

This interview schedule is aligned with the Program Logic Model (PLM) evaluation framework developed for Nepean Blue Mountains Partners in Recovery.

*A brief introduction will be provided. The purpose of the interview and requirements of participation including consent and audio recording, and confidentiality will be reiterated from information provided to the participant earlier. The participant will be given an opportunity to ask further questions and, if ready to proceed, will be given a consent form to sign. Participant information on their representative roles with Partners in Recovery is sought at the commencement of the interview and some demographic information is collected at the end of the interview on a separate sheet.*

Note: “Partners in Recovery” will be referred to in full, rather than abbreviated as “PIR”, within actual interviews

**Could I begin by asking you some preliminary information first please?**

In what capacity is your primary involvement with Partners in Recovery in the Nepean Blue Mountains area?

Community member

Consumer

Carer

Board/ Management/Staff of one of the consorting organisations/partners/agencies

Which stakeholder organisation?

PIR Lead Organisation

Local Health District

Community Clinical Mental Health Team

Personal Helper and Mentors (PHaMS)

Housing and Accommodation Support Initiative (HASI)

Day program provider

Sustenance and short term shelter provider

Housing

Employment

Drug and Alcohol

Disability services

Legal Services

Other (please specify)……………………………………………………………………………………………….

What is your role?

Board

Management

Staff member

Health care provider:

Psychologist

Counsellor

Psychiatrist

General Practitioner

Nurse

Allied health

Other stakeholder (please specify) …………………………………………………………………

Are you are willing to answer some questions where indicated, on behalf of your organisation, health care discipline, or stakeholder group?

Yes

No

| **Topic** | **Lead Question** | **Probe Question** | **Participants** |
| --- | --- | --- | --- |
| 1. Partners in Recovery aim to draw on expertise from across its region. Good relationships with staff and all of its stakeholders are crucial in delivering PIR services | 1.1 Could you please  describe your  relationship with  Partners in Recovery  in NBM? | How long have you been  engaged with Partners in  Recovery? | All Participants |
|  | 1.2 Could you please  describe your  experience interacting  with the Partners in  Recovery in NBM?  (PLM1.4) | -What opportunities do  you have for providing  input to Partners in  Recovery in NBM?  -How do feel about the consultation process? | Consumer reps,  consumers, carers |
| 2. Partners in Recovery seek to demonstrate a robust culture with good management and governance structures | 2.1 What do you know  of Partners in Recovery  management practices?  2.2 How strong do you think these processes are?  2.3 Why or why not?  2.4 How well does Partners in Recovery inform its staff and other stakeholders of its operations? (PLM 1.2) | Please explain? | Partners in Recovery consortium Board, management and staff, other stakeholder staff and management |
| *Additional Question*  **A**. Funding is crucial to Partners in Recovery effectively delivering services | A.1 Could you please  describe how well funding is allocated and dispersed (1.1)? | Is it adequate?  Please explain? | Partners in Recovery consortium Board, management and staff, other stakeholder staff and management |
|  | A.2 How is funding allocated and timed according to program priorities? (PLM1.1) | How do you feel about the funding process? |  |
| 3. The use of technology including information technology underpins the efficient operations of Partners in Recovery | 3.1 How do you find technology and IT support assist in the operations of Partners in Recovery? (PLM 1.6) | -How does the technology assist communications?  -How are you supported to use the technology?  -How satisfied are you with the level of support? | Partners in Recovery consortium Board, staff and management; other stakeholder staff and management |
| *Additional Question*  **B.** It is important that all consortium partners are working together towards a common goal | Could you comment on how  the strategic plans of  NBMML as the Lead Org for  PIR aligns with the strategic  plans of its consortium  partners? (PLM 2.1) | What could tell you that the  strategic plans of all  consortium partners are  shared?  Can you say if this is  happening?  Where/how? | Partners in Recovery  consortium Board, staff and  management; other  stakeholder staff and  management |
| 4. In order for  Partners in Recovery  to best meet the  complex needs of  consumers, an  agenda of  subprograms must be  developed by  consortium partners  focused on a “shared  vision for systems  change” | 4.1 Please tell me about your understanding of “systems change” as Partners in Recovery envisages this.  (PLM 2.1) | What systems change do  you think Partners in  Recovery wants to  achieve? | Partners in Recovery  Consortium staff and  management; other  stakeholder and service  provider staff and  management |

| 5. The success of Partners in Recovery is assisted through broad consultation in program planning and development, as well as through use of clinical and research evidence | 5.1 What is your  experience of  consultation for  planning and  developing Partners in  Recovery? (PLM2.1) | -Could you describe how  you contributed to this  process?  -How satisfied were you with this?  -To what extent do you  think Partners in Recovery  consulted with your  organization/ health  discipline/ stakeholder  group? | Partners in Recovery  consortium and other  service provider  stakeholder staff and  management, consumers  and carers, consumer  reps  Partners in Recovery  consortium and,  other stakeholder staff  and management |
| --- | --- | --- | --- |
|  | 5.2 What do you think  about the use of good  evidence in planning and  developing Partners in  Recovery? (PLM 2.1) | -Where did you see  program planning and  development using  evidence?  -What sort of evidence  was used?  -Was this enough?  -Explain? |  |
| 6. Shared  understandings of  Partners in Recovery  are achieved by using  a common language | 6.1 What is unique about  the language for Partners  in Recovery? (PLM 2.1)  Please explain?  *(Interviewer to clarify if*  *not understood)* | Please tell about your  understanding of:  -“Recovery model”  -“Coordinated care” | Community Reps,  Consortium and other  provider staff and  management |
|  |  |  |  |
| 7. Developing and establishing an “Assessment Intake Tool” is important to effectively identify consumer needs and their recovery plan | 7.1 What has been your role in developing and establishing an assessment intake tool for Partners in Recovery consumers? (PLM 2.2) | -How helpful has this  tool been for assessing  consumer needs and  planning their care?  -Please explain more? | Partners in Recovery  consortium staff and  management |
| 8. A model of care coordination and support must reflect local needs and conditions | 8.1 What has been your role in developing a model for coordinated care and support? (PLM 2.2) | -Please describe this care  model  -How aligned is the model  to local needs and  conditions?  -Please explain more?  -Can you comment on  the effectiveness of the  model in coordinating  care and support?  -Please explain more? | Partners in Recovery consortium and other provider stakeholder staff and management |
| 9. Consumers can arrive in crisis with particularly urgent needs. Staff must be supported to intervene until facilitator staff can be appointed | 9.1. What support can you access to temporarily meet consumer needs in times of crisis? (PLM 2.3) | -Can you describe the  adequacy of that support?  (e.g. how satisfied  were you?  Consumers?) | Partners in Recovery Lead Organisation staff and management |

| 10. The effectiveness of PIR relies on staff and all stakeholders to be well oriented to the overall program and its subprograms, and receive ongoing education and support. | 10.1 What programs of  PIR are you involved  with? | -How well do you  understand the *(these)*  PIR program?  -How well prepared do you feel you are for implementing the *(these)* PIR program? | Partners in Recovery consortium and other provider stakeholder staff and management, consumer reps |
| --- | --- | --- | --- |
|  | 10.2 Could you please  describe the preparation  you received to help you  implement the *(these)*  Partners in Recovery  program ? (PLM 3.1) |  |  |
|  | 10.3 Could you please describe the support and education provided to you? (PLM 2.3, 3.1) | -How helpful has this been to you? -What have been the benefits? -Is the level of education and support satisfactory for you? -Are there areas where more education and support is needed? |  |
| 11. PIR in the NBM area has been developed and resourced according to expected demand and identified consumer needs. | 11.1 Could you comment  on how the level of  consumer intake is  consistent with program  expectations? (PLM 3.1) | -Is NBM PIR meeting  local community  demand?  -Could you explain  more?  -What are the barriers?  -What facilitates  consumer intake and  referral? | Partners in Recovery consortium and other provider stakeholder staff and management |
|  | 11.2 How effective are  the referral pathways  that have been  developed? (PLM 3.1) |  |  |
| 12. PIR program  implementation and  day to day running  occurs in  collaboration with  many stakeholders | 12.1 Could you comment  on your involvement  in the day to day  operations of Partners in  Recovery program in the  NBM area? (PLM 3.1) | -How satisfied are you  with your engagement in  PIR?  -What are the best  aspects for you?  -Where do you  experience difficulties | Consortium and other provider stakeholder staff, consumer reps, consumers and carers |
| 13. As part of its  national reporting  requirements, the  Lead Organisation  for Partners in  Recovery must  provide information  on its effectiveness  at setting up and  starting to  implement PIR | 13.1 Could you comment  on how effective you  think NBM PIR and its  consortium partners  have been in  setting up and  implementing Partners  in Recovery? (PLM 3.1) | -Where has NBM PIR and  the consortium partners  been most effective?  -Least effective?  -What do you think has  influenced this? | Consortium Board,  management and  Staff, other provider  stakeholder staff and  management |
|  | 13.2 Could you comment  on how efficient and  cost effective NBM PIR  is? (PLM 3.1) | -How well is PIR operating  within its means?  -Explain please? | Consortium Board and  management, other  provider stakeholder  management |

| 14. Evaluation of PIR is crucially important to inform future program development and innovation | 14.1 How do you see evaluation activities improving Partners in Recovery in NBM? (PLM 3.2) | -Could you provide some  examples?  -Could you describe your active participation in local monitoring and evaluation of PIR?  -How satisfied are you with the evaluation focus of PIR? | Consortium staff and management, other provider stakeholder staff, consumer reps, consumers and carers |
| --- | --- | --- | --- |
| 15. Partners in Recovery aims to improve community knowledge and awareness of their services | 15.1 As a *(interviewer to*  *state stakeholder group),*  could you describe how  your knowledge of PIR has  changed since the  program commenced?  (PLM 4.1) | -How has this affected  you? (E.g. in the way  you use OR provide  services?)  -How satisfied are you with the information provided? | Consumer reps, consumers and carers, health care and other provider stakeholders |
| 16. The NBM Partners  in Recovery aims to  improve access to PIR  services | 16.1 As a consumer/  carer *(interviewer to*  *specify),* how do you  find you are able to  access the PIR services  you need? (PLM 4.2)  16.2 Are you aware of any change (either improvement or worsening) in access? (PLM 4.2) | -How has this affected  you?  Your family? | Consumers |
|  |  | -How has this affected  you?  -The person for  whom you are caring?  -Their family? | Carers |
| 17. The purpose of Partners in Recovery is to make a positive difference to the functioning of all stakeholders | 17.1 How do you regard  PIR as a model that  coordinates support for  people with severe and  persistent mental illness  and complex needs?  (PLM 4.3) | -Is it effective?  - Why or why not?  - Could you provide an example? | Consortium and other  provider stakeholder  staff and management |
|  | 17.2 As a consumer/  carer *(interviewer to*  *specify),* how has your  health and level of,  functioning changed as  a result of PIR? (PLM  4.3) | -Where/how has this  occurred?  -How has this changed  your life?  -How has the level of  burden you feel changed?  -How has this impacted  your family? | Consumers and carers |
|  | 17.3 As a provider of  services, how have your  skills and level of  functioning changed?  (PLM 4.3)  17.4 Can you comment on your engagement in new and effective partnerships that assist your provision of care? (PLM 4.3) | -Where/how has this  occurred?  -What has improved?  -What do you think has  assisted or blocked  improvement?  - How satisfied are you  with these aspects of  PIR so far? | Consortium and other provider stakeholder staff |

| 18. Partners in  Recovery aims to  leave a lasting legacy  of improved health  and wellbeing in its  local community | 18.1 Can you comment  as to whether your  access to the right  services has improved?  -Has this improvement  been maintained over  time? (PLM 5.1) | -How well does PIR meet  your particular needs?  (Are there areas that  could be covered  better)? | Consumers and carers |
| --- | --- | --- | --- |
|  | 18.2 As a service  provider, how has  Partners in Recovery  helped you meet the  care needs of your  consumers? (PLM 5.1) | -How effective is your  provider network in  meeting complex needs  of particular consumers?  -Are there still gaps in  care provision?  -Can you please  describe these? | Consortium and other  provider stakeholder  staff |
| 19. Partners in  Recovery aims to  achieve large scale  “systems change”  through integrated  and coordinated  services | 19.1 How has PIR assisted  your engagement with  other services to provide  a “shared” response to  consumer needs? (PLM  5.2) | -How well are you able to  engage with the right  partners to provide a  shared response?  -What services have been  well engaged?  -Which have not been as  well engaged?  -Are there barriers in  coordinating and  integrating services?  Explain?  -Where does Partners in  Recovery work best?  -Where is there work to  be done? | Consortium and other  provider stakeholder  staff |
|  | 19.2 How well focused do  you think service  providers are operating  based on a “recovery”  model of care? (PLM 5.2) | -Could you provide  examples of where this is  occurring?  -Are there areas where  the recovery focus can be  strengthened? |  |
|  | 19.3 As a consumer/  carer or service  provider, *(interviewer*  *to specify),* can you  comment on improved  access to stable  housing, and  participation in  employment,  education and social  activities? (PLM 5.2) | What role has Partners in Recovery played in working on these aspects of consumer care? | Consumers and carers  and service providers |

**Participants will be thanked for their contribution and asked a final question:**

Is there anything we might have missed or something you would like to add?

**End of interview**

**Participants will be asked if they would also provide demographic information on a separate sheet:**

What is your age group?

18-30

31-50

>51

Are you of Aboriginal or Torres Strait Islander origin?

No

Yes, Aboriginal

Yes, Torres Strait Islander

Both

In which country were you born?

Australia

Other (Please specify)………………………………………..

Do you speak a language other than English at Home? *(If more than one language indicate the one spoken most often)*

No, English only

Yes, other (please specify)……………………………………………………………
