# Supplementary material for: Partners in Recovery: an early phase evaluation of an Australian mental health initiative using program logic and thematic analysis
Source: BMC Health Serv Res. 2019 Jul 26;19:524. doi: 10.1186/s12913-019-4360-2 (PMC6660922; doi:10.1186/s12913-019-4360-2)

**Additional File 5**

**Qualitative coding frame**

The following table of free nodes indicates the sources (number of interview transcripts) and the references (numbers of interview excerpts) corresponding to each node and subnode.


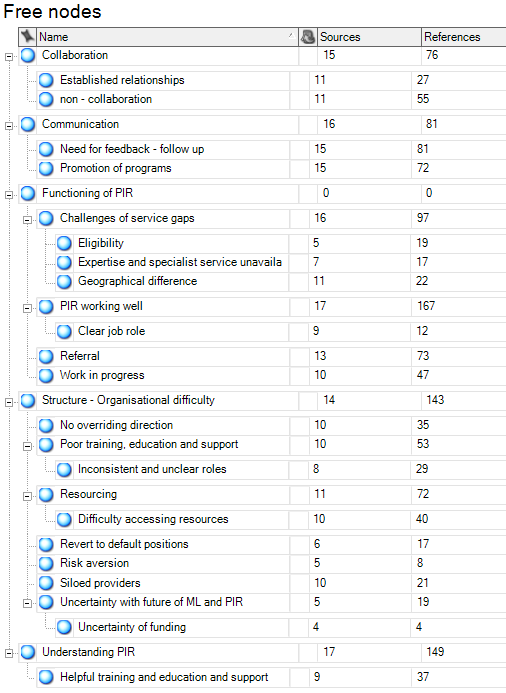

Supplement: Supplementary file 5 — Qualitative coding frame (DOCX 61 kb) [file 12913_2019_4360_MOESM5_ESM.docx]
